# SCOPmap: Automated assignment of protein structures to evolutionary superfamilies

**DOI:** 10.1186/1471-2105-5-197

**Published:** 2004-12-14

**Authors:** Sara Cheek, Yuan Qi, S Sri Krishna, Lisa N Kinch, Nick V Grishin

**Affiliations:** 1 Howard Hughes Medical Institute, University of Texas Southwestern Medical Center, 5323 Harry Hines Blvd., Dallas, Texas 75390, USA; 2 Department of Biochemistry, University of Texas Southwestern Medical Center, 5323 Harry Hines Blvd., Dallas, Texas 75390, USA

## Abstract

**Background:**

Inference of remote homology between proteins is very challenging and remains a prerogative of an expert. Thus a significant drawback to the use of evolutionary-based protein structure classifications is the difficulty in assigning new proteins to unique positions in the classification scheme with automatic methods. To address this issue, we have developed an algorithm to map protein domains to an existing structural classification scheme and have applied it to the SCOP database.

**Results:**

The general strategy employed by this algorithm is to combine the results of several existing sequence and structure comparison tools applied to a query protein of known structure in order to find the homologs already classified in SCOP database and thus determine classification assignments. The algorithm is able to map domains within newly solved structures to the appropriate SCOP superfamily level with ~95% accuracy. Examples of correctly mapped remote homologs are discussed. The algorithm is also capable of identifying potential evolutionary relationships not specified in the SCOP database, thus helping to make it better. The strategy of the mapping algorithm is not limited to SCOP and can be applied to any other evolutionary-based classification scheme as well. SCOPmap is available for download.

**Conclusion:**

The SCOPmap program is useful for assigning domains in newly solved structures to appropriate superfamilies and for identifying evolutionary links between different superfamilies.

## Background

Protein structure classifications are commonly used for studying structural and evolutionary relationships between proteins (namely remote homology inference), protein structure and function prediction, identification of potential functional residues and binding sites, understanding sequence/structure/function relationships in proteins, and as an aid in describing protein folds and families.

Several structural classification schemes such as SCOP [1], CATH [2], and Dali Domain Dictionary [3] have been developed for the purpose of cataloguing all available protein structures. These databases are commonly used for studying structural and evolutionary relationships between proteins. Detecting remote homology between protein structures is a difficult task because of the challenge in differentiating between distant homologs and structural analogs. Several researchers have reported the inadequacy of various structural similarity measures for distinguishing homologous and analogous relationships [4-7]. Therefore, although the databases mentioned above are associated with automatic methods for identifying potential structural neighbors of a new protein query, they are often incapable of assigning domains to a unique position in the classification according to evolutionary relationships. Determining appropriate evolutionary relationships within a database is usually accomplished by expert manual analysis. Although manual classification of protein structures remains the gold standard, the necessity for reliable automatic tools that can reproduce the results of such a classification scheme becomes increasingly apparent as available databases continue to grow in size. Such tools must be capable of detecting homology between distantly related proteins while keeping false positives at a minimum.

Available tools for assigning proteins to existing classification schemes use either structure-based or sequence-based comparison methods. Classification predictions from structure comparison tools like SSM [8], GRATH[9], and F2CS [10] are generally accurate to the fold or topology level but do not necessarily have evolutionary implications. Consequently, establishing homology between the query and the predicted neighbors often requires a more thorough examination. Classification assignments from sequence comparison tools such as SUPERFAMILY [11] can detect homology but often miss the more remote homologous relationships suggested by structural similarities. These tools are generally reliable for homology detection in easy to moderate cases but frequently produce many false positive results for more distant relationships. A strategy combining information from both sequence and structure comparisons would be expected to perform better than either method alone by exploiting the advantages of each approach.

In this paper, we describe an algorithm developed to map domains within protein structures with their homologs in an existing classification scheme. The general strategy employed by this algorithm is to combine the results of several existing sequence and structure comparison tools in order to determine classification assignments. The comparison tools incorporated in the algorithm each utilize a different methodology for identifying homologous domains, and consequently, these tools have different advantages and limitations. An approach combining different methods of homology detection is expected to capitalize on the proficiencies of each comparison tool while the limitations of those tools are neutralized by the inclusion of other methods.

Our algorithm, named SCOPmap, has been developed to map domains in protein structures to the SCOP database, which is a manually curated hierarchical classification scheme based on the structural and evolutionary relationships between proteins. SCOPmap assigns protein domains at the superfamily level, which is the broadest level of homology in the SCOP database. SCOPmap also performs assignments at the SCOP fold level when confident superfamily level assignments cannot be made. SCOPmap has two general applications. First, domains within newly solved protein structures can be identified and assigned to the appropriate SCOP superfamily. Second, SCOPmap can be used to find new links in SCOP by identifying potential evolutionary relationships between existing SCOP superfamilies. The strategy employed by this algorithm is not limited to SCOP and could be applied to any other similar database or classification scheme as well.

We have evaluated the performance of SCOPmap on two test sets, each of which includes over 4500 protein domains. The first set is comprised of the proteins that are included in SCOP v1.63 but not in SCOP v1.61, while the second set contains the proteins that are included in SCOP v1.65 but not in SCOP v1.63. SCOPmap was able to correctly map greater than 94% of both test sets at the SCOP superfamily level. Comparison of SCOPmap results and SUPERFAMILY [11] results for the same test set indicates that SCOPmap performs better than SUPERFAMILY both in terms of overall correct assignments and in accurate definition of the domain boundaries of those assignments. We have analyzed SCOPmap's performance at both the SCOP superfamily and SCOP fold levels. We have also evaluated the performance of the individual comparison tools incorporated in the algorithm. Furthermore, we describe examples of difficult cases that are successfully mapped and investigate the reasons why some domains are not mapped automatically by our algorithm.

## Results

### Evaluation of SCOPmap performance

#### Mapping of the tweaking set domains

Results of SCOPmap performance on the tweaking set are shown in Table 1 (see Methods for description of tweaking and testing sets). Correct SCOP superfamily assignments were made for 87.8% of the tweaking set domains. For an additional 0.3% of the tweaking set domains, the superfamily assigned by SCOPmap is not the same as the SCOP-assigned superfamily. However, in each of these cases, the superfamily assigned by SCOPmap and the superfamily specified by SCOP are homologous. For example, SCOPmap assigns the 7-bladed β-propeller domain of an archael surface layer protein to a homologous SCOP superfamily of 6-bladed β-propellers [12]. Because the purpose of the SCOPmap is to assign domains at the broadest level of homology in the classification (i.e. the SCOP superfamily level), such cases are not considered false positives but instead reflect special cases in the SCOP database. 6.2% of the tweaking set domains were given no superfamily assignment by SCOPmap, but are domains that belong to SCOP superfamilies that are new in v1.63. Because such domains cannot be appropriately assigned to a superfamily that is represented in the library used by SCOPmap (v1.61 in this case), these are also considered correctly mapped (i.e. true negative assignments). Thus, a total of 94.3% of the tweaking set domains are correctly mapped by SCOPmap. The remaining 5.7% of the tweaking set are false negative assignments. These domains belong to superfamilies that exist in SCOP v1.61, but no superfamily assignment is made by SCOPmap.

#### Mapping of the testing set domains

Results of SCOPmap performance on the testing set (see Methods) are shown in Table 1. Correct SCOP superfamily assignments were made for 91.2% of the testing set domains. In an additional 0.2% of the test set, the domain assignments given by SCOPmap are homologous to the superfamilies specified by SCOP. 3.1% of the tweaking set domains are given true negative assignments. These are cases in which the appropriate superfamily assignment is not a part of the library used by SCOPmap (based on SCOP v1.63 in this case), and no superfamily assignment is made by SCOPmap. Thus, a total of 94.5% of the testing set domains are given correct assignments by SCOPmap. 5.3% of the testing set domains are false negative assignments in which the domain belongs to a superfamily that is present in SCOP v1.63, but no superfamily assignment is made by SCOPmap. The remaining 0.2% of the testing set domains are given false positive assignments.

#### False positive assignments in the testing set

Because the score cutoffs used by SCOPmap's individual comparison tools were determined while considering domains from the tweaking set, those cutoffs were therefore influenced by the specific collection of domains in that set. Had a different test set been considered when establishing these cutoffs, it is likely that the score cutoffs would be slightly different. Thus, the few false positive assignments observed in the second test set are not unexpected. Furthermore, the number of false positive domain assignments made is higher than the number of incorrect hits between query and library domains that are accepted. Due to redundancy in the test set (e.g. often one structure contains several identical chains and therefore several identical domains), the 7 domains mapped incorrectly essentially reflect only 3 different examples of false positive assignments.

Each incorrectly assigned domain has less than 10% sequence identity to the nearest library representative from the same SCOP superfamily. Furthermore, all of the false positive assignments are due to scores from the individual comparison tools which barely meet the cutoffs required for acceptance. Such cases reflect the influence that a few specific domains can have in determining the exact values of the minimum score threshold requirements. All incorrect assignments were made due to a hit accepted by one of the comparison tools that includes both sequence and structure components.

For example, addiction antidote protein MazE from *Escherichia coli *(PDB code: 1mvf, chains D and E[13]; SCOP domains: d1mvfd_ and d1mvfe_) belongs to the Kis/PemI addiction antidote superfamily in SCOP and forms a pseudobarrel as a homodimer. SCOPmap incorrectly maps this protein to the "Transcription-state regulator AbrB, the N-terminal DNA recognition domain" superfamily in SCOP, which is a 2-layer α/β protein. This assignment is due to a hit found to the N-terminal DNA recognition domain of AbrB from *Bacillus subtilis *(PDB code: 1ekt [14]; SCOP domain: d1ekta_). Although the aligned regions of these two domains have the same secondary structure (an α-helix, a β-strand, and followed by a β-hairpin) and similar spatial arrangement, the overall topologies of these folds are highly dissimilar. This hit is accepted due to the 18 pairs of residues from the query and library representative which are equivalently aligned in pairwise alignments produced by PSI-BLAST (E-value = 55) and DaliLite (Z-score = 0.2). As the score cutoffs required by this comparison tool are E-value ≤ 100, Z-score > 0, and number of equivalent residue pairs ≥15, this particular query-library hit clearly falls just within the boundaries of the accepted score ranges.

The nuclease domain of putative ATP-dependent RNA helicase Hef from *Pyrococcus furiosus *(PDB codes: 1j22, 1j23, 1j24, and 1j25 [15]; SCOP domains: d1j22a_, d1j23a_, d1j24a_, and d1j25a_), a member of the restriction endonuclease-like superfamily in SCOP, is incorrectly mapped to the FAD/NAD(P)-binding domain superfamily. This assignment is made because of a conservation pattern analysis hit to NADH-dependent ferredoxin reductase BphA4 from *Pseudomonas *strain KKS102 (PDB: 1d7y [16]; SCOP domain: d1d7ya2). Although the core of both the query and the library representative is an α/β domain containing a 5-stranded β-sheet, the overall topology is not similar. This query-library pair hit is accepted because of the matrix-based conservation score of 0.32, which is based on the structural alignment of these two domains by DaliLite (Z-score = 3.7), while the score cutoffs required by this comparison tool are matrix-based score ≥ 0.25 and DaliLite Z-score ≥ 2. Again, the scores for this hit fall near the boundaries of the accepted score ranges.

The proteolytically-cleaved peptide C from bovine lysosomal α-mannosidase (PDB code: 1o7d [17]; SCOP domain: d1o7d.2) belongs to the galactose mutarotase-like superfamily in SCOP, but is incorrectly mapped to the "alpha-Amylases, C-terminal domain β-sheet domain" superfamily. This assignment is due to a hit identified by conservation pattern analysis to the C-terminal domain of neopullulanase from *Bacillus stearothermophilus *(PDB code: 1j0h [18]; SCOP domain: d1j0ha2). Although the core of lysosomal α-mannosidase peptide C and the C-terminal domain of neopullulanase each form a β-sandwich-like fold, the topologies of these folds are different. The COMPASS-based conservation score for this query-library pair (0.52) is based on the structural alignment of the two domains by DaliLite (Z-score = 4.6). These scores fall just within the required ranges for acceptance by the conservation pattern comparison method (COMPASS-based conservation score ≥ 0.5 and DaliLite Z-score ≥ 2).

#### Comparison of tweaking and testing set results

Table 1 shows that the SCOPmap results are comparable for the tweaking set and the testing set. SCOPmap performance on the two test sets are nearly equivalent: 94.3% (tweaking set) *vs *94.5% (testing set) correct assignments; 5.7% (tweaking set) *vs *5.3% (testing set) false negative assignments; and 0.0% (tweaking set) *vs *0.2% (testing set) false positive assignments. The most significant apparent differences are in the results for the specific types of correct assignments: true positives with ranges accurate within 10 residues, true positives with ranges that are not accurate within 10 residues, and true negatives. These seemingly disparate results are predominantly reflections of inconsistencies in test set composition rather than in SCOPmap performance. More specifically, these variations are primarily due to the number of query domains that belong to new SCOP superfamilies. The most obvious consequence is the fraction of each test set given true negative assignments (6.2% in tweaking set, 3.1% in testing set), which is directly dependent on the fraction of each test set that belongs to new SCOP superfamilies. If domains from new SCOP superfamilies are ignored, the apparent disparity in SCOPmap boundary definition accuracy is reduced. For example, if the entire test sets are considered, there is a 2.3% difference in the number of domains correctly assigned whose ranges are accurate within 10 residues of the SCOP-defined boundaries. However, when considering only domains that can potentially be mapped correctly (i.e. domains that do not belong to new SCOP superfamilies), 86.8% of the tweaking set domains are correct assignments that are accurate within 10 residues, compared to 86.4% of the testing set domains. Similarly, 92.4% of all correctly assigned domains in the tweaking set are accurate within 10 residues, compared to 91.6% for the corresponding domains in the testing set.

The comparable results are a reliable indication of the consistency of SCOPmap performance because the two test sets are of nearly equivalent difficulty. First, the two test sets include approximately the same fraction of trivial assignments: 73.7% of mappable domains in the tweaking set are assigned by gapped BLAST while 73.6% of mappable domains in the testing set are assigned by gapped BLAST, where "mappable" means the domain is both evolutionarily relevant and is a member of a SCOP superfamily that exists in the version of SCOP used as the library. Of the non-trivial mappable domains (i.e. mappable domains that are not assigned by gapped BLAST), the average sequence identity between the query domain and the closest library representative from the same SCOP superfamily is 29.2% in the tweaking set and 28.6% in the testing set.

#### Fold level assignments

**Figure 1:**
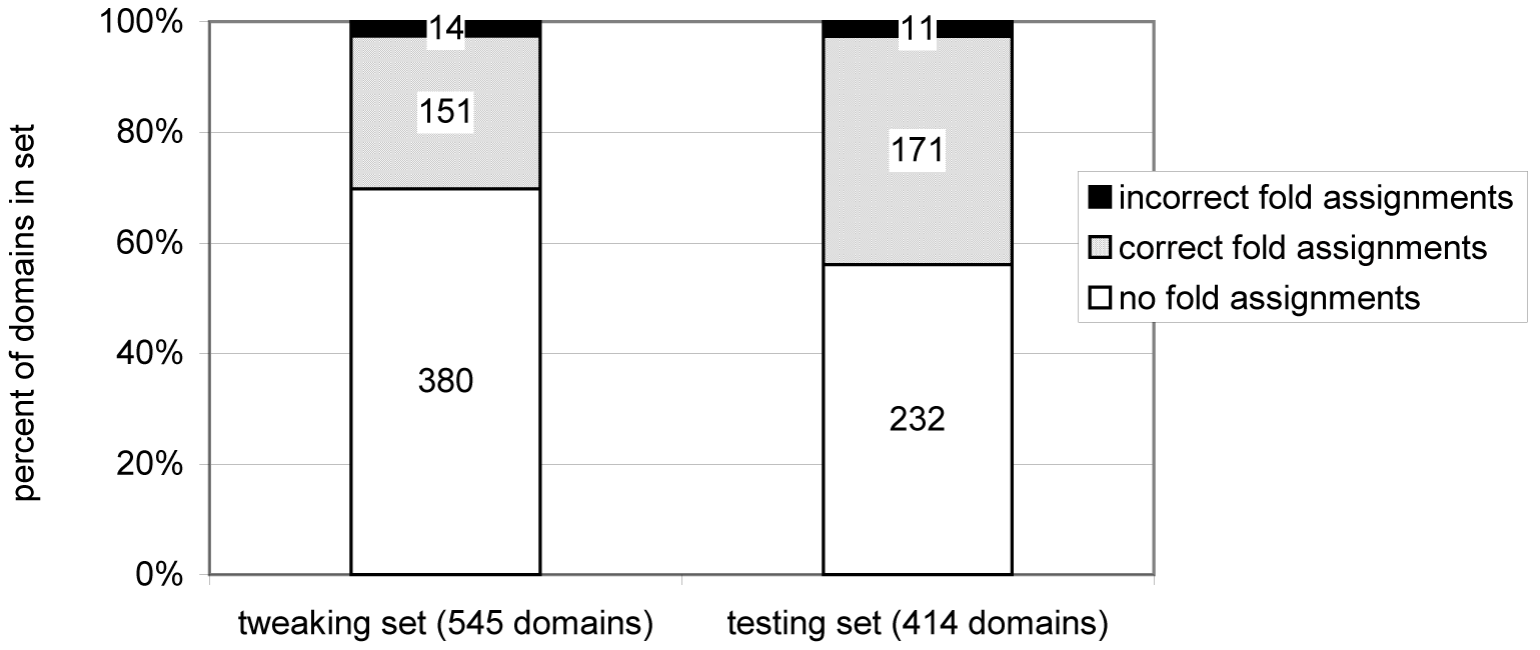
*Fold level assignments*.

Fold level assignments are attempted for regions of query chains at least 20 residues in length for which no superfamily assignment was made. Results are shown in Figure 1. In the tweaking set (v1.61-v1.63 test set), fold level assignments are made for ~30% of the 545 SCOP-defined domains with no superfamily level assignment. 92% of these fold level assignments are correct. In the testing set (v1.63-v1.65 test set), fold level assignments are made for ~44% of the 414 SCOP-defined domains with no superfamily level assignment. Of these assignments, ~94% are correct.

Similar to the superfamily level assignments, the apparent disparity in fold level assignments are due primarily to the relative composition of the two test sets rather than inconsistency in performance. There are two principal attributes of test set composition that result in improved fold level results. First, domains from new folds are typically given no fold level assignment by SCOPmap, so a smaller fraction of unmapped domains from new folds will result in a decreased number of domains for which no assignment is made. Second, because the structural similarity between two domains from the same superfamily is likely to be greater than that between two domains from different superfamilies within the same fold, a larger fraction of unmapped domains from existing superfamilies will result in an increased number of correct fold level assignments. Both of these attributes favor the testing set over the tweaking set (results not shown). This indicates that the testing set is less challenging in terms of fold level assignments, which is consistent with the improved results relative to the tweaking set (Figure 1).

Although no fold level assignment is made in a large number of cases (~70% of tweaking set unmapped domains and ~56% of testing set unmapped domains), this result is not altogether unexpected for several reasons. First, as discussed above, a significant fraction of the unmapped domains in each set belong to new SCOP folds, so no appropriate fold level assignment exists among the set of library representatives. Next, the minimum Z-score cutoff required for making fold level assignments is strict in order to minimize false positive assignments. While Ortiz *et al*. report that MAMMOTH Z-scores greater than 5.25 are generally reliable for fold predictions [19], we find that a MAMMOTH Z-score of 10 is required for making reliable fold assignments. Although 45% of domains in the tweaking set from existing folds but without a fold assignment (171 of 380 domains) have at least one MAMMOTH hit to a representative of the appropriate fold with a Z-score between 5.25 and 10, results in this range are not used due to many occurrences of false positive assignments (data not shown). Conversely, because MAMMOTH Z-scores greater than 22 are sufficient for assignments at the superfamily level (see Methods), fold assignments are neither necessary nor made for query-library domain pairs with such overwhelming structural similarity. Furthermore, because query-library domain pairs with sufficient sequence similarity to be recognized by automatic methods are mapped at superfamily level, unmapped domains have very little sequence similarity to the corresponding library representatives. Consequently, fold assignments are made only for a rather limited set of queries: domains with extremely low sequence similarity as well as significant but not overwhelming structural similarity to library representatives.

The false positive rates are nearly identical in the two test sets (~2.6%). In both sets, the false positive rate of fold level assignments is significantly higher for domains that belong to new SCOP folds compared to those from existing SCOP folds. For example, in the second testing set, 6 of the 86 domains that belong to new folds have incorrect fold level assignments (7.0%) while only 5 of the 328 domains from existing folds are given an incorrect assignment (1.5%). Because false positive hits are likely to fall just above the Z-score cutoff for fold level assignment, many false positives are ignored due to other hits found with better Z-scores, which are true positives in most cases. Thus, because domains that belong to existing SCOP folds should have significant structural similarity to at least one library domain (i.e. the library representative(s) of that particular SCOP fold), the negative effect of false positive hits to these domains is minimized in the false positive rate relative to that for domains from new SCOP folds.

False positive fold level assignments are typically due to a query and library representative sharing similar but not identical topology. For example, the structure of riboflavin kinase (PDB code: 1n06 [20]; SCOP domain: d1n06b_) is a query in v1.61-v1.63 test set and belongs to a SCOP superfamily that is new to SCOP v1.63. Appropriately, no superfamily level assignment is made. The fold of riboflavin kinase is a n = 6, S = 10 β-barrel with strand order 163452, but SCOPmap assigns this domain to the double psi β-barrel fold in SCOP, which is an n = 6, S = 10 β-barrel with strand order 163425. In this case, the incorrect fold assignment is based on similarity of overall topology, but other false positive fold assignments occur when a region within a query domain and a region within a SCOP representative have similar topology despite overall dissimilarity of the folds. For example, the structure of the ε-subunit of the plasmid maintenance system (PDB code: 1gvn [21]; SCOP domain: d1gvna_) is another query in v1.61-v1.63 test set which also belongs to a new superfamily in SCOP v1.63. Again, no superfamily level assignment is made, as appropriate. The fold of the ε-subunit is a 3-helix up-and-down bundle with left-handed twist, but SCOPmap assigns this domain to a 4-helix up-and-down bundle fold. The three α-helices in the query domain and the last three α-helices of the SCOP representative have identical topology, similar lengths, and equivalent spatial orientation to each other. This false positive is a result of the query topology matching a region of a SCOP representative. The opposite case, when a region of the query domain is the same as the topology of an entire SCOP representative, occurs as well. For example, the structure of viral chemokine binding protein m3 (PDB code: 1mkf [22]; SCOP domain: d1mkfa_), a query in v1.61-v1.63 test set, belongs to a new fold in SCOP v1.63. Appropriately, no superfamily level assignment is made for this query. The fold of this domain is a 10-stranded β-sandwich with 6 β-strands in one sheet and 4 in the other. This domain is mapped at the fold level to an 8-stranded β-sandwich with 4 β-strands in each sheet. Although the overall folds of these two domains are different, 7 β-strands from each of these two β-sandwich folds have identical topology and mutual spatial arrangement.

Unsurprisingly, correct fold assignments are made predominantly for typical globular proteins while no fold assignments are made for small protein or coiled coil folds. Outside of this observation, there are no recognizable trends suggesting types of folds for which assignments are more easily made.

Furthermore, it should be noted that fold assignments are not our main goal. Rather, these assignments are a by-product of the comparison tools that are used for mapping at the superfamily level by SCOPmap. The purpose of making fold level assignments is merely to assist the user in further study of those domains which SCOPmap does not assign at the superfamily level. The fold level mapping strategy and score cutoffs have not been optimized to perform fold mapping with high sensitivity or low false positives.

### Performance of SCOPmap compared to SUPERFAMILY

#### Overall performance

SUPERFAMILY is another tool that attempts to assign domains within a query protein to the superfamily level of SCOP. It is the only package that we are aware of that meets our two requirements for direct comparison: the program performs a similar task and is available for download. The results of the performance of SUPERFAMILY relative to SCOPmap are shown in Table 1. Overall, SCOPmap performs better than SUPERFAMILY. SUPERFAMILY correctly maps 91.4% of domains compared to the 94.3% assigned to the correct SCOP superfamily by SCOPmap. Furthermore, SCOPmap is much more proficient at defining accurate domain boundaries. SCOPmap delineates domain boundaries within 10 residues of the SCOP-defined boundaries for 81.4% of domains, while SUPERFAMILY performs as well in only 70.1% of cases. This difference is due partly to the use of MAMMOTH and DaliLite in our algorithm. However, the results of our algorithm when using only sequence comparison tools show that there is still a 6.5% advantage over SUPERFAMILY in terms of accurately defined ranges (Table 1). Thus, the inclusion of structure comparison methods is not solely responsible for the dramatic improvement in boundary definition. Presumably, a second predominant factor in the increased domain boundary accuracy is the strict coverage criteria for sequence comparison methods incorporated in SCOPmap.

Table 1 shows the results of using only the BLAST, RPS-BLAST, PSI-BLAST, and COMPASS portions of our algorithm. This modified version of SCOPmap (henceforth referred to as the "sequence-only algorithm") was expected to perform similarly, if not better than, SUPERFAMILY. It was therefore surprising to observe significantly more false negative assignments by the sequence-only algorithm compared to the SUPERFAMILY algorithm (12.5% and 8.6%, respectively). Investigation of the 573 false negatives from the sequence-only algorithm indicates three general explanations for these missed assignments (data not shown). In ~47% of these cases (270 of 573 domains), there are no sequence comparison hits below the required E-value thresholds. Next, in ~17% of cases (97 of 573 domains), sequence hits that pass both the E-value and coverage criteria are found, but the domain is not assigned due to an unresolved choice between conflicting superfamilies. In the remaining 36% of cases (206 of 573 domains), sequence comparison hits to at least one superfamily representative are found that pass the required E-value cutoffs but fail the coverage criteria. These 206 domains correspond to ~4.5% of this test set and account for the difference in false negative rates between the sequence-only algorithm and SUPERFAMILY, which does not have a coverage requirement.

#### Performance on non-trivial domain assignments

Because nearly 70% of the domains can be mapped using only gapped BLAST (Table 3), the results of both SCOPmap and SUPERFAMILY are skewed in favor of trivial domain assignments. In order to evaluate the performance of these two programs on more challenging assignments, the results were re-tabulated excluding all domains assigned via gapped BLAST (Table 2). Here, SCOPmap assigns 81.6% of domains to the appropriate SCOP superfamily while SUPERFAMILY correctly maps 77.1% of domains, so SCOPmap's advantage in correctly assigned domains increases from 2.9% for all domains to 4.5% for only non-trivial assignments. SCOPmap's proficiency in domain boundary definition is also accentuated, as the difference in percent of domains with accurately defined domain boundaries increases from 11.3% for all domains (SCOPmap: 81.4%, SUPERFAMILY: 70.1%) to 12.8% for non-trivial assignments (SCOPmap: 42.8%, SUPERFAMILY: 30.0%). Thus, evaluating only the non-trivial assignments emphasizes the advantages of SCOPmap over SUPERFAMILY.

#### Comparison of false negative assignments

The false negative assignments made by SCOPmap (261 domains) and by SUPERFAMILY (395 domains) were compared in order to determine the degree of overlap between the two sets of unassigned domains. One might expect that a significant number of the false negative assignments would be shared by the two algorithms and would represent those cases that are too difficult to be confidently mapped by existing automatic comparison tools. Indeed, 205 domains are given false negative assignments by both SCOPmap and SUPERFAMILY.

Therefore, of the 261 false negative assignments made by SCOPmap, only 56 domains (21%) are correctly mapped by SUPERFAMILY. 38 of these domains were correctly identified by at least one of the comparison methods used but were not assigned (due, for example, to an unresolved choice of superfamily assignment). Most of the remaining domains that were assigned by SUPERFAMILY but not identified by SCOPmap represent cases that are typically difficult for automatic methods: 8 are small disulfide-rich domains, 3 are relatively short domains (74, 75, and 126 residues) that are interrupted by very large insertions (290, 289, and 282 residues respectively), and 1 domain contains many short breaks in the sequence and structure. The few remaining examples are domains that could have been reasonably expected to be mapped by SCOPmap: *E. coli *succinate dehydrogenase subunit SdhC (PDB codes: 1nek [23], chain D and 1nen [23], chain D; SCOP domains: d1nekd_ and d1nend_) is a helical bundle protein that belongs to the succinate dehydrogenase/fumarate reductase transmembrane segment superfamily in SCOP, and the PKD-like domain of *Methanosarcina mazei *surface layer protein (PDB codes: 1l0q [12], chains A, B, C, and D; SCOP domains: d1l0qa1, d1l0qb1, d1l0qc1, d1l0qd1) is an immunoglobulin-like domain that belongs to the PKD domain superfamily in SCOP. Other than the low sequence identity between these queries and the library representatives of the corresponding SCOP superfamilies, there are no convincing arguments for why these assignments might not be made. In each of these cases, significant hits are found by the structure comparison tools used in SCOPmap: SdhC has a DaliLite Z-score of 8.7 to a library representative of its SCOP superfamily, and surface layer protein PKD-like domain has a MAMMOTH Z-score of 10.6 to the library representative of its SCOP superfamily. However, the limited sequence similarity between the query and representative domains results in insufficient BLOSUM scores to meet the required score cutoffs of these methods. Although these are consequently false negative assignments at the superfamily level, the correct fold level assignment was made in each of these last 6 cases.

Conversely, approximately half of the false negative assignments made by SUPERFAMILY (190 of 395 domains) are correctly mapped by SCOPmap. Of these domains, ~54% are first identified by a sequence comparison tool in SCOPmap (gapped BLAST, RPS-BLAST, PSI-BLAST, or COMPASS), ~29% are first identified by a structure comparison tool (MAMMOTH or DaliLite), and the remaining ~17% are first identified by a method that combines both sequence and structure information (correlation of conservation patterns or the agreement of DaliLite alignments with gapped BLAST, RPS-BLAST, or PSI-BLAST alignments).

## Discussion

### Performance of individual comparison methods

In order to assess the relative performance of the individual comparison tools used by SCOPmap, the number of assignments in the tweaking set gained by each additional comparison method was evaluated. The results are summarized in Table 3. For each comparison tool, the number of domains first identified by that method was determined, and the percent of previously unassigned domains gained by that method was calculated. The comparison tools are listed in order of increasing sensitivity to distant homologs: sequence comparison methods (BLAST, RPS-BLAST, PSI-BLAST, and COMPASS), structure comparison methods (MAMMOTH and DaliLite), and finally comparison methods that incorporate both sequence and structure information (correlation of conservation patterns and agreement of DaliLite alignments with BLAST, RPS-BLAST, or PSI-BLAST alignments). Domains are included in the total count for only the least sensitive comparison tool that identified the hit.

**Figure 2:**
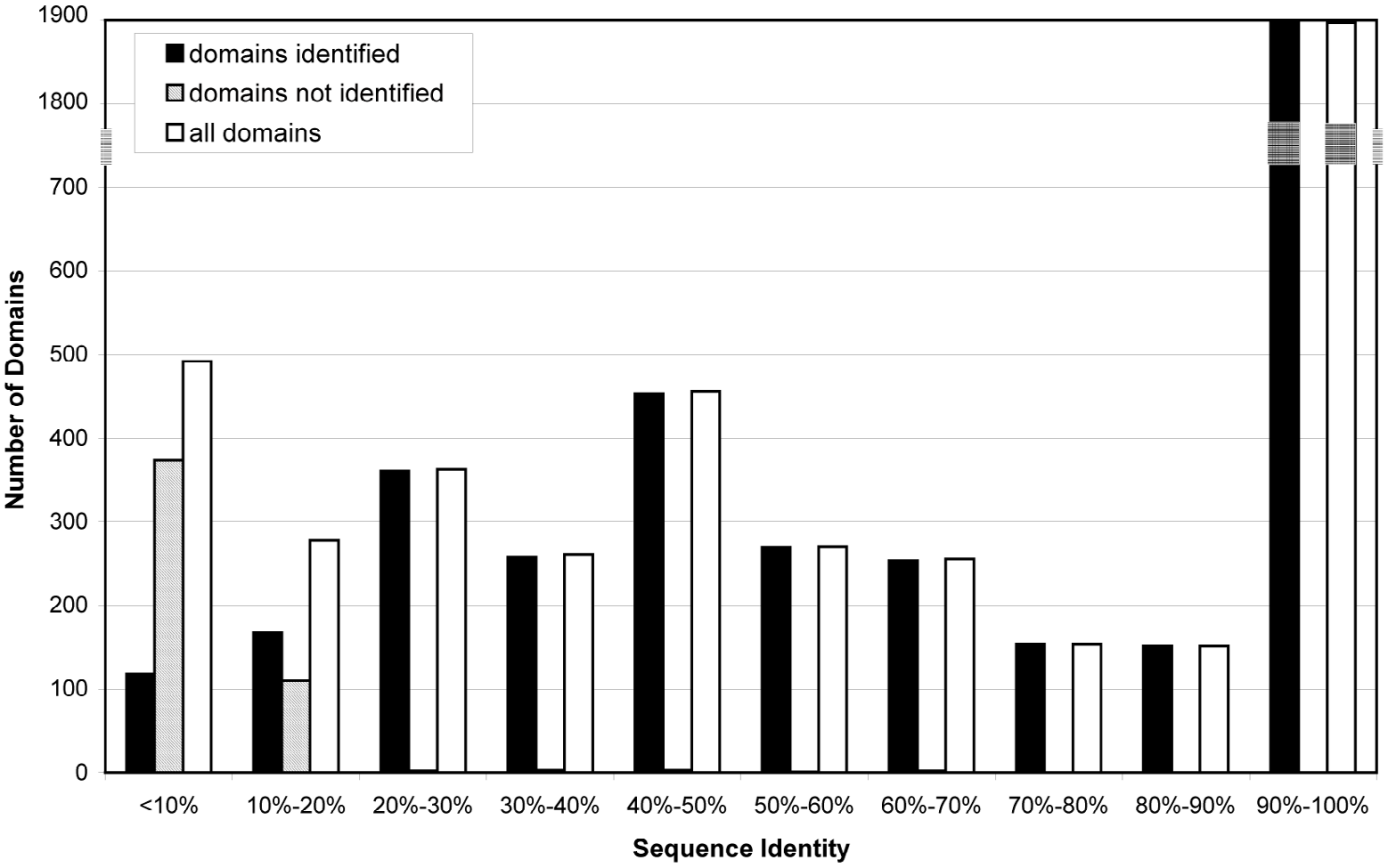
*Sequence identity between tweaking set domains and the closest library representative from the same SCOP superfamily*.

The most number of assignments are made by gapped BLAST and RPS-BLAST, which give 69.1% gain and 36.3% gain of previously unmapped domains, respectively. However, these assignments are among the easiest in the set. The average sequence identity between the query domain and the closest library representative of that superfamily is 80.1% for gapped BLAST assignments and 41.1% for RPS-BLAST assignments. Furthermore, these numbers are considerably inflated as a consequence of the surfeit of trivial assignments in the tweaking set (Figure 2).

PSI-BLAST, MAMMOTH, and DaliLite each give between 10% and 20% gain of previously unmapped domains. The average sequence identities between the identified query domains and the library domains indicate that these assignments are neither trivial nor unusually difficult. The two structure comparison methods show similar overall performance by this assessment, although DaliLite does have the advantage over MAMMOTH both in number of assignments and percent gain as well as in difficulty of assignments made. This seemingly implies that comparison via MAMMOTH is an unnecessary step, and indeed, nearly all domain assignments made by MAMMOTH are also made by DaliLite (data not shown). However, MAMMOTH is both necessary for and proficient at determining potential hits by DaliLite. The pre-identification of potential hits drastically reduces the running time compared to comprehensive comparison of the query domains to all library domains by DaliLite. Furthermore, MAMMOTH is essential for making fold level assignments.

The conservation pattern analysis and the calculation of agreement between DaliLite alignments and BLAST, RPS-BLAST, or PSI-BLAST alignments have 4.2% and 5.9% gain of previously unmapped domains, respectively. Although the numbers of additional assignments are among the lowest of any of the comparison tools, these two methods also make the most challenging assignments of any of the comparison tools included in SCOPmap. The average sequence identity between query domains and library representatives for assignments made first by these methods is less than 15%. Specific examples are discussed below.

Thus, the general observation is that, as expected, those comparison tools more sensitive to distant homology typically make more challenging assignments, but with lower percent gains. The only clear exception to this trend is COMPASS. COMPASS has the lowest percent gain of any step at 3.3%, and the domains first identified by this method are only moderately difficult assignments (average sequence identity 27.2%). This is presumably due in part to the extremely strict E-value cutoff necessary for avoiding false positives (1 × 10^-10^). Furthermore, of the four sequence comparison tools used in SCOPmap, COMPASS is most sensitive to remote homologs. Therefore, if the query-library domain pair has sufficient sequence similarity to be recognized by automatic methods, it is likely that the hit would also be identified by one of the less sensitive sequence comparison tools and consequently be accounted for earlier in Table 3.

### SCOPmap performance on remote homologs

#### Correctly mapped remote homologs

The similarity of the tweaking set to the representative library domains is shown in Figure 2 (white bars). Nearly 50% of tweaking set domains are more than 70% identical to one of the library representatives from the same SCOP superfamily. Furthermore, 69.1% of the tweaking set domains can be correctly mapped by gapped BLAST (Table 3). Other domains, however, are more difficult to assign due to limited similarity of the query domain to the representative library domains. SCOPmap is able to make several such assignments, including nearly 300 domains with less than 20% sequence identity to the closest library domain from the same SCOP superfamily (black bars, Figure 2).

**Figure 3:**
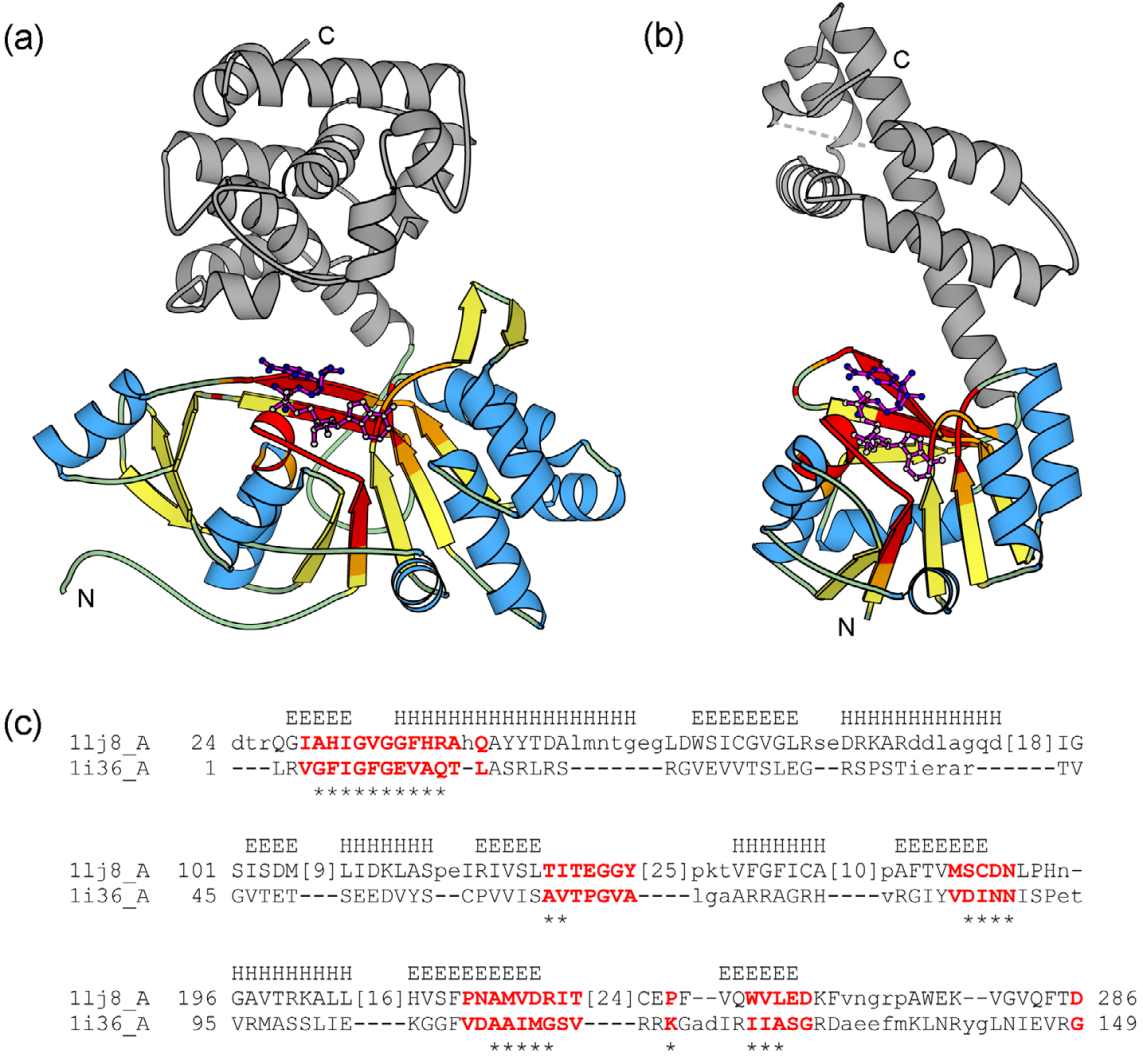
***Correctly mapped remote homolog: N-terminal domain of mannitol 2-dehydrogenase***. a) Ribbon diagram of mannitol 2-dehydrogenase from *Pseudomonas fluorescens *(PDB: 1lj8 [19]). The N-terminal domain is shown in color. Regions in red are positions among the top 25% of most conserved positions in both the query (1lj8_A, N-terminal domain) and library representative (1i36_A, N-terminal domain). Regions in orange are positions among the top 25% of most conserved positions in either the query or the library representative domain, but not both. Positions in this domain that are not among the most highly conserved are blue (α-helices), yellow (β-strands), and green (coils). The C-terminal domain is shown in grey, and the bound nucleotide in shown in ball-and-stick format and is colored magenta. This and all other structure figures were prepared using MOLSCRIPT[48]. b) Ribbon diagram of conserved hypothetical protein MTH1747 from *Methanobacterium thermoautotrophicum *(PDB: 1i36). The N-terminal domain, shown in color, is a representative of the NAD(P)-binding Rossmann-fold superfamily. The colors have equivalent meaning to those in figure 3a. The C-terminal domain is shown in grey, and nucleotide in shown in ball-and-stick format and is colored magenta. Dashed lines indicate breaks in the chain. c) Pairwise alignment of the query (1lj8_A) and library representative (1i36_A) from DaliLite results. Residues in red bold text are among the top 25% of most conserved positions in at least one of the domains. Residues indicated with an asterisk are among the top 25% of most conserved positions in both the query and library domains. Secondary structure is indicated above the alignment, with E signifying β-strand residues and H signifying α-helix residues. In this and other alignments, the numbers flanking the alignment indicate the residue number in the sequence of the first (or last) aligned residue on that line. Numbers in brackets specify the number of residues in an insert that are not shown. In all alignments produced based on DaliLite results, capital letters are aligned residues and lower-case letters are unaligned residues.

One prevalent difficulty in making classification assignments by automatic methods is correctly assigning domains that have very limited sequence similarity to the library representatives. One such example of a difficult but correctly assigned domain is the N-terminal domain of mannitol 2-dehydrogenase from *Pseudomonas fluorescens *(PDB code: 1lj8 [19], N-terminal domain; SCOP domain: d1lj8a2). In SCOP, this domain belongs to the NAD(P)-binding Rossmann-fold domains superfamily. There are 90 representatives of this superfamily in the library, all of which have less than 10% sequence identity to the query domain. There are no BLAST, RPS-BLAST, PSI-BLAST, COMPASS, MAMMOTH, or DaliLite hits to these library representatives that pass both the required coverage and E-value or Z-score thresholds. Hits to three of the 90 superfamily representatives are identified by DaliLite: the N-terminal domain of glycerol-3-phosphate dehydrogenase from *Leishmania mexicana *(PDB code: 1evy [24], N-terminal domain; SCOP domain: d1evya2) with Z-score 6.9, the N-terminal domain of conserved hypothetical protein MTH1747 from *Methanobacterium thermoautotrophicum *(PDB code: 1i36 [25], N-terminal domain) with Z-score 6.3, and the N-terminal domain of lactate/malate dehydrogenase from *Methanococcus jannaschii *(PDB code: 1hye [26], N-terminal domain; SCOP domain: d1hyea1) with Z-score 6.4. Because of the poor BLOSUM scores calculated for the pairwise alignments given by DaliLite, none of these hits are accepted by the DaliLite comparison method. However, these relatively high Z-scores indicate that the DaliLite alignments are reliable enough for use in the comparison of conservation patterns method, and hits to two of these superfamily representatives are accepted based on correlation of conservation patterns: the N-terminal domain of glycerol-3-phosphate dehydrogenase (SCOP domain: d1evya2) has matrix-based conservation score = 0.26, and the N-terminal domain of conserved hypothetical protein MTH1747 (SCOP domain: d1i36a2) has matrix-based conservation score = 0.11. In both of these cases, approximately 75% of the most conserved positions in the query domain and in the library domain are equivalent (Figure 3c). Furthermore, these most conserved positions are clustered around the nucleotide-binding sites, which are equivalent in these domains (Figure 3a,b). The N-terminal domain of this query structure is therefore mapped to the NAD(P)-binding Rossmann-fold domain superfamily in SCOP based on the high degree of correlation between the conservation patterns of the query domain and these two superfamily representatives.

**Figure 4:**
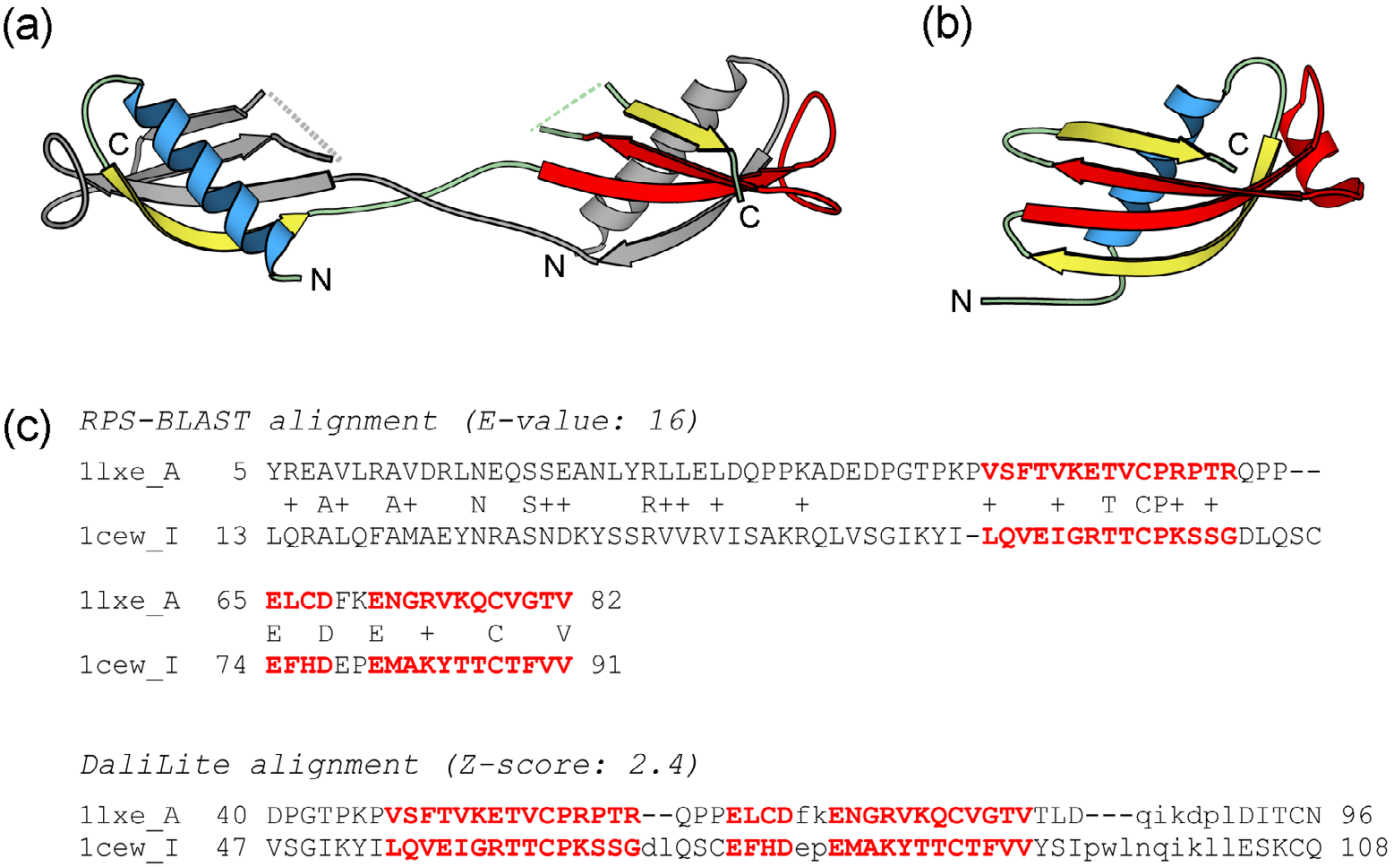
***Correctly mapped domain with conformational variation: cathelicidin motif of protegrin-3***. a) Ribbon diagram of cathelicidin motif of protegrin-3 from *Sus scofa *(PDB: 1lxe[27]) in a swapped dimer conformation. One monomer in the complex is colored, and the second monomer is grey. Chain breaks are indicated by dashed lines. b) Ribbon diagram of cystatin from *Gallus gallus *(PDB: 1cew[28]), a library representative of the cystatin/monellin superfamily. c) Pairwise alignments of this query (1lxe_A) and library (1cew_I) domain produced by RPS-BLAST and DaliLite. Residues aligned equivalently by these two comparison tools are in red bold. The equivalently aligned regions are shown in red in the structure figures.

Conformational differences between similar protein domains also result in challenging classification assignments for automatic structure comparison tools. One such example is the antimicrobial cathelicidin motif of protegrin-3 from *Sus scofa *(PDB code: 1lxe [27]; SCOP domain: d1lxea_). The crystal structure of this protein shows the domain in a swapped dimer conformation (Figure 4a). The closest library representative to this query domain is cystatin from *Gallus gallus *(PDB code: 1cew [28]; SCOP domain: d1cewi_), which belongs to the cystatin/monellin superfamily in SCOP. This domain is a monomer in the crystal structure (Figure 4b). The sequence identity between the query (cathelicidin motif of protegrin-3) and this library representative (cystatin) is approximately 19%. The hit between the query and this library representative is found by both the RPS-BLAST and DaliLite methods. However, the scores for these hits are relatively poor as a result of the low sequence identity and the conformational variation between the two domains. The scores for these comparisons (RPS-BLAST E-value = 16 and DaliLite Z-score = 2.4) fail the score cutoff criteria for these methods individually. Comparison of the alignments produced by these two methods, however, indicates that a significant portion of the domain is aligned equivalently by RPS-BLAST and DaliLite (Figure 4c). Thus, based on the agreement of these two methods, the cathelicidin motif of protegrin-3 is correctly mapped to the cystatin/monellin superfamily of SCOP.

**Figure 5:**
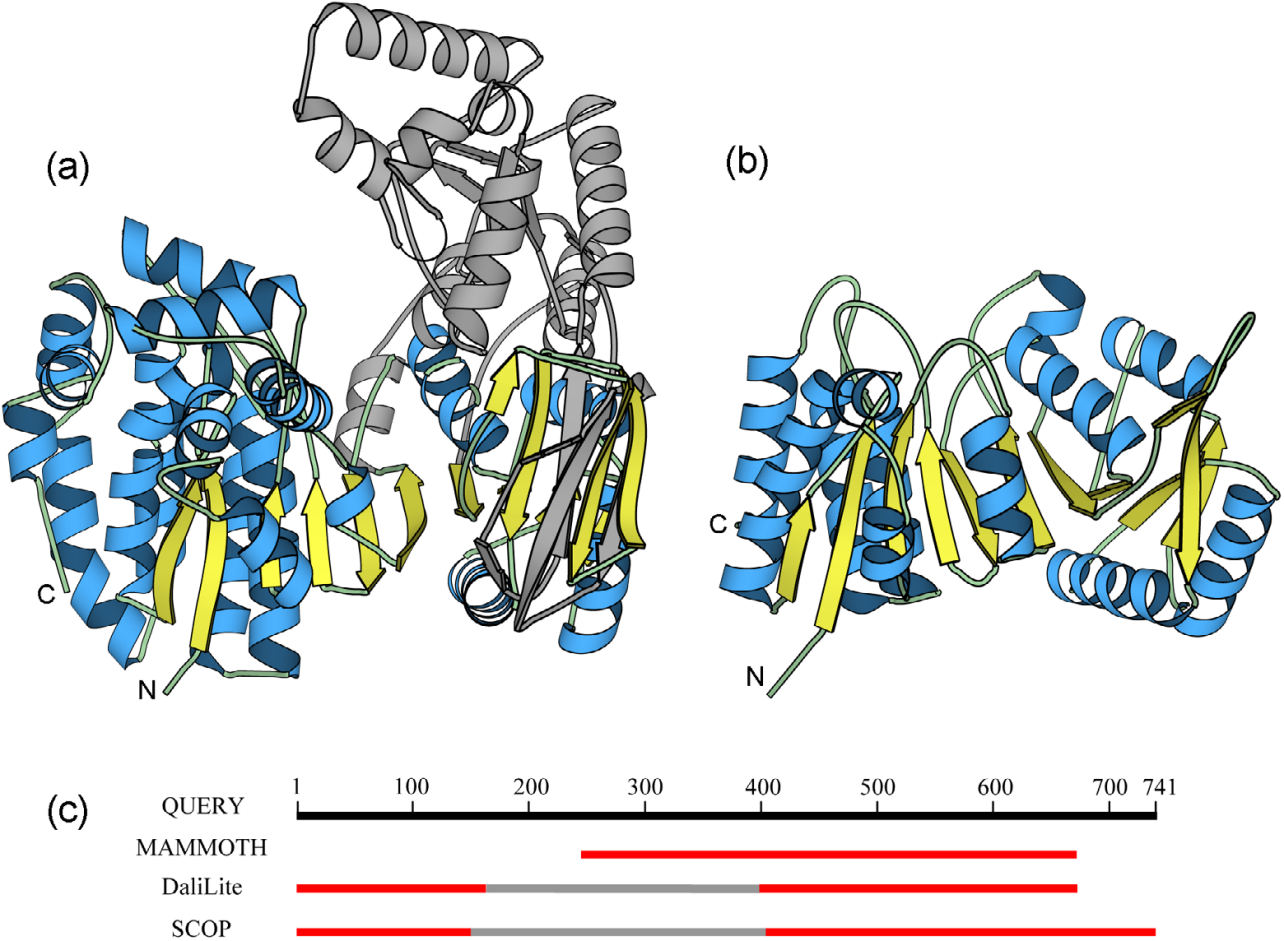
***Correctly mapped domain with large insertion: monomeric isocitrate dehydrogenase***. a) Ribbon diagram of monomeric isocitrate dehydrogenase from *Azotobacter vinelandii *(PDB: 1itw[29]). The insert region as defined by SCOP is shown in grey. b) Ribbon diagram of isopropylmalate dehydrogenase from *Salmonella typhimurium *(PDB: 1cnz[30]), a library representative of the isocitrate/isopropylmalate dehydrogenase superfamily. c) Range assignments as made by MAMMOTH, DaliLite, and SCOP. The regions assigned to the isocitrate/isopropylmalate dehydrogenase superfamily are red and insert regions are grey.

Another common problem for many automatic comparison methods is the presence of large insertions or deletions in the query domain. This third example demonstrates the ability of the mapping program to correctly assign such cases. Monomeric isocitrate dehydrogenase from *Azotobacter vinelandii *(PDB code: 1itw [29]; SCOP domain: d1itwa_) belongs to the isocitrate/isopropylmalate dehydrogenase superfamily in SCOP. There are two representatives of this superfamily in the library, both of which have less than 15% sequence identity to the query domain. Furthermore, the query domain has an approximately 250-residue insertion relative to the superfamily representatives (Figure 5). There are no BLAST, RPS-BLAST, PSI-BLAST, or COMPASS hits to either library representative. Although the MAMMOTH hit to 3-isopropylmalate dehydrogenase from *Salmonella typhimurium *(PDB code: 1cnz [30]; SCOP domain: d1cnza_) is accepted with Z-score 22.2, the presence of the large insertion in the query results in an erroneous range definition by MAMMOTH (Figure 5c). Comparison of the query to this same library representative by DaliLite identifies residues 164–397 as an insertion in this domain (Figure 5c). Although SCOP assigns the entire chain of monomeric isocitrate dehydrogenase as one domain (residues 1–741), residues 150–404 are defined as an insert region. Thus, the DaliLite-based assignment made by SCOPmap (residues 2–163, 398–671) is a reasonably accurate domain definition.

#### Domains without SCOPmap assignments at the superfamily level

In 5.7% of the tweaking set, no superfamily assignment is made for domains that should belong to superfamilies that are included in SCOP v1.61. General explanations for these false negative assignments are summarized in Table 4. Of the 261 unmapped domains, 19.2% percent (50 domains) are found by meeting the required score cutoffs of one or more of the comparison tools used, but these domains are not assigned due to a conflict with another domain identified in the same query chain. There are two ways in which this may happen: there may be an unresolved choice of superfamily assignment over a certain region of the query chain, or the boundary of one domain may erroneously extend over a second domain resulting in one domain being assigned while the another domain is missed.

In the remaining 80.8% of unmapped domains, comparison of the query to the library domains do not pass the score cutoffs of any of the methods used. These domains typically have only limited structural similarity as well as less than 20% sequence identity to the library representatives. All domains that have greater than ~20% sequence identity to a library representative from the same SCOP superfamily but are not identified by any of the comparison tools used in SCOPmap are small protein domains less than 50 residues in length. Because automatic methods often perform poorly on small proteins, such cases are not unexpected. These unmapped small protein examples comprise only 0.2% of the tweaking set. Furthermore, the unmapped domains often have inserted or deleted structural elements relative to the library domains. The unmapped and unidentified domains fall into three general categories in terms of structural similarity to the library representatives. First, 33.3% of unmapped domains have very little structural similarity to the corresponding library domains. When the MAMMOTH scores for a query domain are insufficient for making superfamily assignments, these scores are used as an initial indicator of whether specific query-library domain pairs are likely to be assigned by DaliLite (see Methods). For these unmapped domains, the MAMMOTH scores to library domains are too poor to be identified even as potential hits. Next, there are a small number of cases (6.1% of unmapped domains) that have potential but unconfirmed structural similarity to library representatives. In these cases, one or more potential hits are identified by MAMMOTH, but DaliLite does not produce output for those pairs. This could mean that the DaliLite Z-score is less than zero for the given pair of domains, or that either the query domain, the library representative, or both could not be handled by DaliLite because, for example, the structure lacks recognizable secondary structure, contains only Cα coordinates, or is less than 30 residues in length, *etc*. Finally, the remaining 41.4% of unmapped domains have recognizable but insufficient structural similarity to the library representatives. For these domains, hits are found via DaliLite but the scores of the hits do not meet the required cutoffs. Because such scores cannot be confidently distinguished from false positives, no superfamily assignment is made.

Since the inception of the SCOP database, the rapid growth in the number of available protein structures has resulted in a classification scheme that is not equally uniform in all parts. This is primarily apparent in overpopulated folds and superfamilies, such as TIM β/α-barrels, where intermediate relationships exist but are difficult to describe within the original SCOP classification scheme. These special cases in the SCOP database also contribute to the rate of false negative assignments by SCOPmap. In a later section, the conservative nature of SCOP is demonstrated by cases in which homologous proteins are assigned to different superfamilies. As a consequence of this attribute of the SCOP database, good hits via automatic comparison methods are sometimes found to multiple SCOP superfamilies. In some cases, SCOPmap is not capable of selecting one final assignment out of several correct choices. These 28 examples, which make up the unresolved choice of superfamilies category in Table 4, account for less than 1% of the tweaking set but 10.7% of all false negative assignments. Conversely, there are also numerous instances in which the SCOP classification is quite liberal. Examples are rampant in the sections of the database that the authors describe as not a part of the proper SCOP classification, such as the low resolution structures and peptides classes. These classes are not included in the SCOPmap library and are therefore not considered by our algorithm. However, cases were also observed in the evolutionarily relevant multi-domain proteins class of SCOP. The multi-domain proteins class is problematic in the sense that it deviates from the format followed by the remainder of the SCOP database. Members of this class have not been classified at the domain level, and there is often wide variation in the size and domain composition of the entries. One such example was detected during the manual investigation of false negative assignments from the tweaking set. Reovirus polymerase λ3 (PDB code: 1n1 h [31]; SCOP domain: d1n1ha_) belongs to the DNA/RNA polymerases superfamily in the multi-domain proteins class of SCOP. The structural fold of domains in the DNA/RNA polymerases superfamily has been described as a "right-hand" configuration containing "palm", "fingers", and "thumb" subdomains. Domains in this superfamily, of which there are >200, typically include 2 or 3 subdomains of the "right-hand" fold. For example, Moloney murine leukemia virus (MMLV) reverse transcriptase (PDB code: 1mml [32]; SCOP domain: d1mml__), which is one of the representatives of this superfamily included in the v1.61 library, is a 265-residue fragment containing only the "palm" and "fingers" subdomains. Reovirus polymerase λ3, however, also includes a 380-residue N-terminal domain as well as a 377-residue C-terminal "bracelet" domain, in addition to the "palm", "fingers", and "thumb" subdomains. Thus, a 1267-residue, 3-domain protein (reovirus polymerase λ3) and a 265-residue, single domain fragment (MMLV reverse transcriptase) are classified equivalently at the superfamily level in SCOP. Naturally, such variations within the database are problematic for making appropriate classifications via automatic methods.

#### Examples of false negative SCOPmap assignments

**Figure 6:**
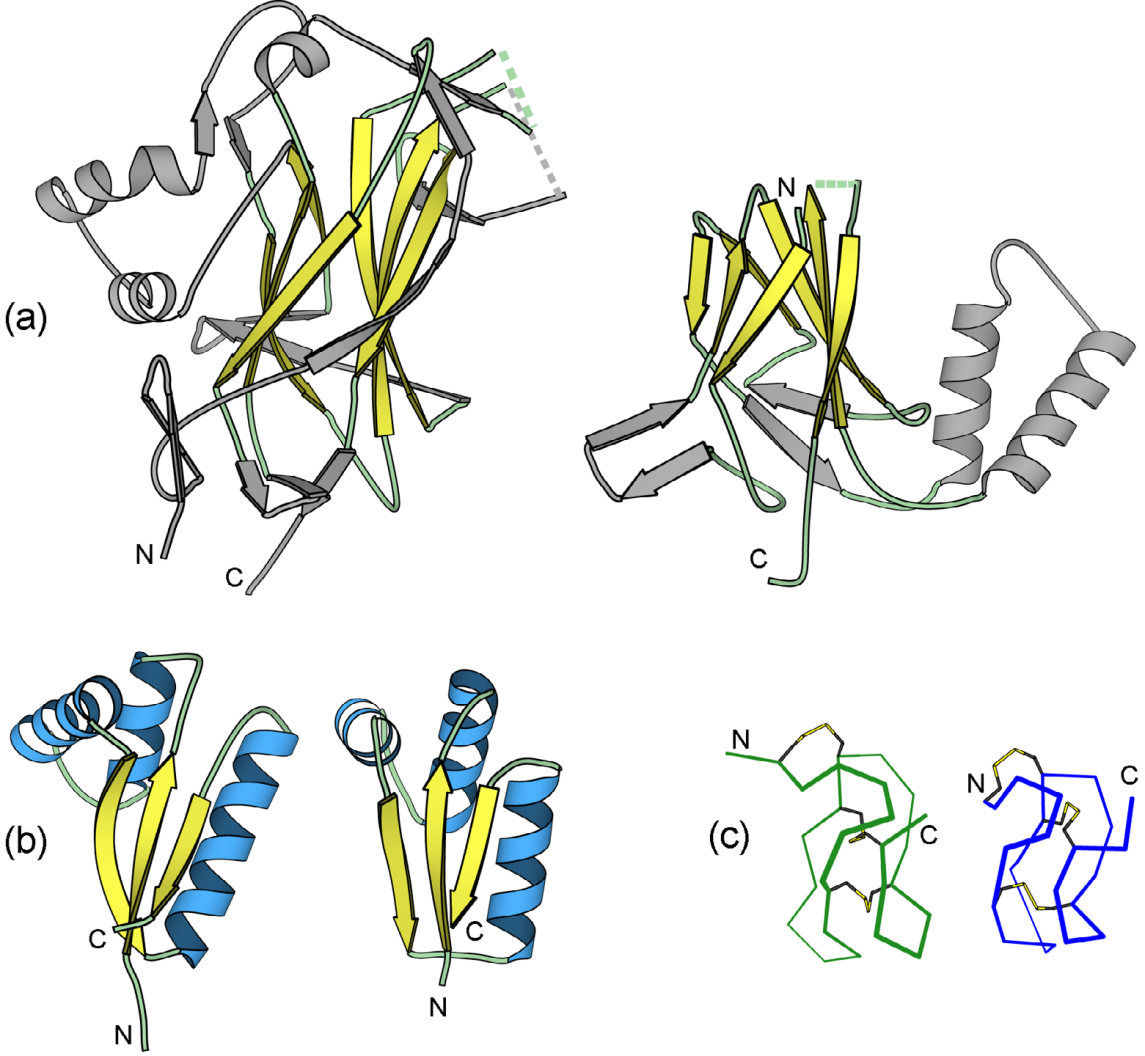
***Examples of false negative domain assignments***. a) Ribbon diagrams of unmapped domain (left) transcription factor Ndt80 (PDB: 1mnn[33]) and library representative (right) p52 subunit of NF-kappa B, N-terminal domain (PDB: 1a3q[49], residues A37-A226). β-strands that belong to the Ig fold core are yellow, and additional structural elements are grey. Dashed lines indicate breaks in the chain. b) Ribbon diagrams of unmapped domain (left) *E. coli *adaptor protein ClpS (PDB: 1lzw[34], chain A) and library representative (right) ribosomal protein L7/12 from *E. coli*, C-terminal domain (PDB: 1ctf[50]). Dashed lines indicate breaks in the chain. c) Cα traces of unmapped domain (left) δ-conotoxin TxVIA from *Conus textile *(PDB: 1fu3[35]) and library representative (right) ω-conotoxin TXVII from *Conus textile *(PDB: 1f3k[51]). These two conotoxin domains share ~40% sequence identity. Disulfide bonds are shown in ball-and-stick format.

Some superfamily assignments are missed due to extremely limited similarity between the query domain and the corresponding library representatives. One such example is *Saccharomyces cerevisiae *DNA-binding domain from transcription factor Ndt80 (PDB code: 1mnn [33]; SCOP domain: d1mnna_), which belongs to the p53-like transcription factors superfamily in SCOP. Members of this superfamily bind DNA through an s-type Ig fold. There are seven library representatives of this superfamily, all of which have less than 10% sequence identity with the query domain. There are no hits to these representatives found by BLAST, RPS-BLAST, or PSI-BLAST with E-value less than 100 or by COMPASS with E-value less than 1 × 10^-3^. Because the MAMMOTH hits to these representatives are very poor (Z-scores below 2.5), MAMMOTH finds neither accepted hits nor potential hits for comparison via DaliLite. Although the conserved core of this superfamily is observable by eye (Figure 6a), the many inserted structural elements relative to the library representatives contribute to the poor performance of the automatic structural comparison methods. The DNA-binding function of this domain may have contributed to its inclusion in this superfamily by the SCOP authors.

Superfamily assignments are also missed in cases where the similarity to library representatives is moderately significant but still insufficient for distinction from false positives. One such example is adaptor protein ClpS from *E. coli *(PDB code: 1lzw [34], chain A; SCOP domain: d1lzwa_) (Figure 6b), which belongs to the ClpS-like superfamily in SCOP. The one representative of this superfamily in the library shares ~11% sequence identity with the query domain. BLAST, RPS-BLAST, and PSI-BLAST hits to this library representative are not found with E-values less than 100, and a COMPASS hit to the library domain is not found with E-value less than 1 × 10^-3^. Comparison of the query and library domain by MAMMOTH and DaliLite give more substantial results: a MAMMOTH Z-score of 10.4 with BLOSUM score -1.0 × 10^-2 ^for the pairwise alignment produced by MAMMOTH, and a DaliLite Z-score of 8.8 with BLOSUM score 4.5 × 10^-4 ^for the pairwise alignment produced by DaliLite. Unfortunately, these scores fall just below the required cutoffs for superfamily assignment via these methods. Thus, no superfamily assignment is made. However, the MAMMOTH Z-score does meet the fold level cutoff, so a correct fold assignment is made for this query domain.

Additionally, technical shortcomings of automatic methods contribute to missed superfamily assignments. For example, δ-conotoxin TxVIA from *Conus textile *(PDB code: 1fu3 [35]; SCOP domain: d1fu3a_) is a 27-residue small protein that belongs to the omega toxin-like superfamily in SCOP. There are 21 library representatives of this superfamily, some of which share up to 40% sequence identity with the query domain. However, there are no hits to these representatives found by BLAST, RPS-BLAST, or PSI-BLAST with E-value less than 100 or by COMPASS with E-value less than 1 × 10^-5^. The MAMMOTH hits to these 21 representatives all have Z-scores well below 4. Furthermore, DaliLite cannot handle this protein due to the short length, thus precluding DaliLite comparisons with library representatives. Thus, despite significant sequence and structural similarity of δ-conotoxin TxVIA to several library representatives (Figure 6c), no superfamily assignment is made due to the poor performance of automatic methods on small proteins.

### Finding new links between SCOP superfamilies: examples of homologs in different SCOP superfamilies identified by SCOPmap

**Figure 7:**
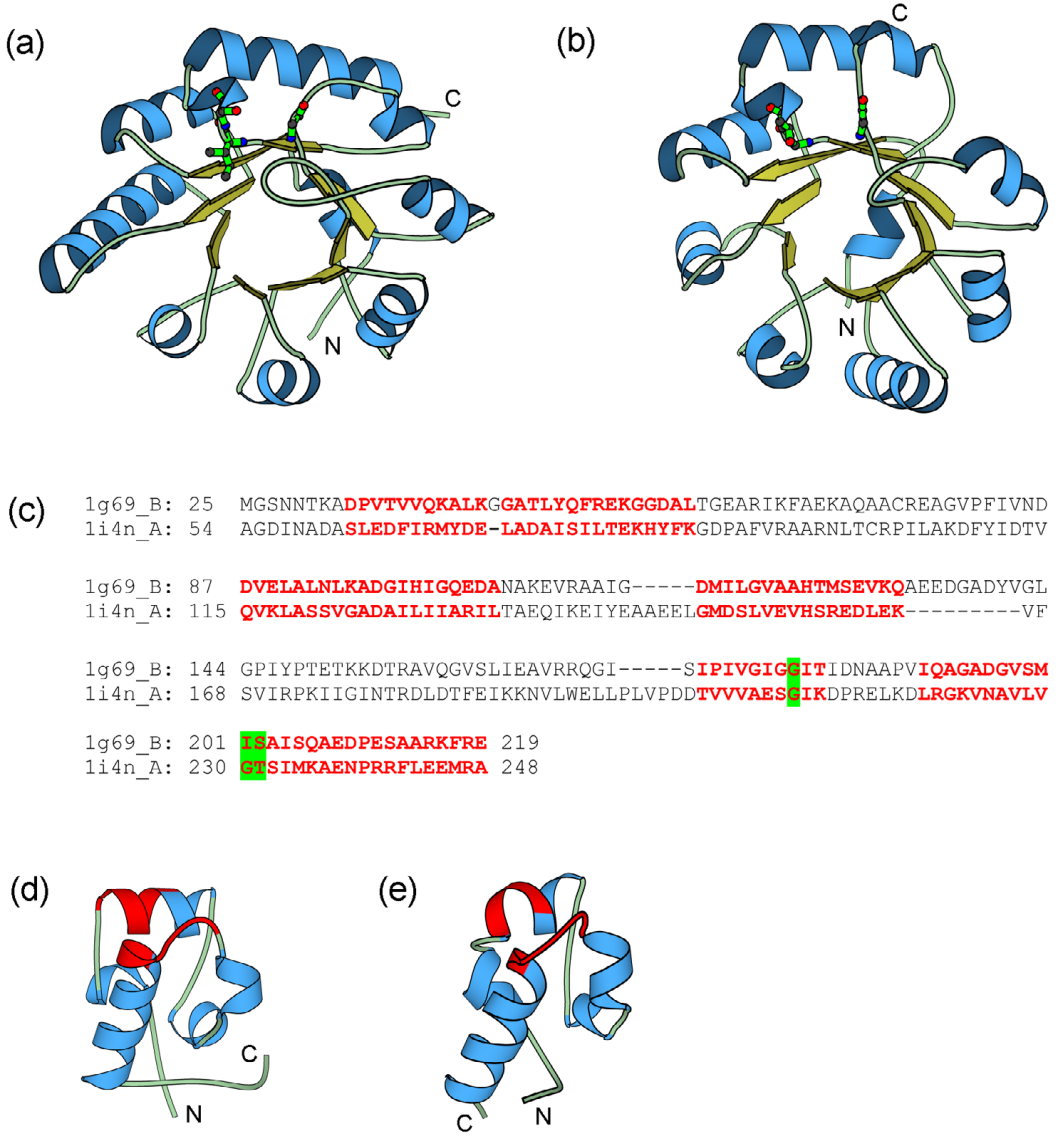
***Homologous SCOP superfamilies***. a) Ribbon diagram of thiamin phosphate synthase from *Bacillus subtilis *(PDB: 1g69[52], chain B) from the thiamin phosphate synthase superfamily. b) Ribbon diagram of indole-3-glycerophosphate synthase from *Thermotoga maritima *(PDB: 1i4n[53], chain A) from the ribulose-phosphate binding barrel superfamily. c) Pairwise alignment of representatives of the thiamin phosphate synthase and the ribulose-phosphate binding barrel superfamily produced by PSI-BLAST. Residue pairs that are equivalently aligned by DaliLite are showed in red bold letters. The three phosphate-binding residues in conserved positions in these two proteins are highlighted in green in the alignment and shown in ball-and-stick format in the structure figures. d) Ribbon diagram of α subunit C-terminal domain from *E. coli *RNA polymerase (PDB: 1lb2[54], chain B) from the "C-terminal domain of RNA polymerase alpha subunit" superfamily. Regions of the domain involved in DNA binding are in red. e) Ribbon diagram of the N-terminal domain of Rad51 from *Homo sapiens *(PDB: 1b22[55], chain A) from the "DNA repair protein Rad51, N-terminal domain superfamily". Putative DNA-binding surface is in red.

The thiamin phosphate synthase superfamily and the ribulose-phosphate binding barrel superfamily are one example of homologous SCOP superfamilies identified by SCOPmap. Both superfamilies have a TIM β/α-barrel fold. When thiamin phosphate synthase is used as the query, hits to 8 different members of the ribulose-phosphate binding barrel superfamily are identified. These hits are found by PSI-BLAST, COMPASS, DaliLite, and the agreement between pairwise alignments produced by DaliLite and by RPS-BLAST or PSI-BLAST. Because confident hits are identified by both sequence and structure comparison methods, the homology between the two superfamilies is considered reliable, despite the limited sequence identity (<20%). The structure of thiamin phosphate synthase and indole-3-glycerophosphate synthase, which is a representative of the ribulose-phosphate binding barrel superfamily, are shown in Figure 7a,b. The RPS-BLAST alignment (E-value 1 × 10^-10^) (Figure 7c) and the DaliLite alignment (Z-score 15.4) of these two proteins are similar: 101 pairs of residues (~40% of the proteins) are equivalently aligned by the two comparison tools. Furthermore, three phosphate-binding residues are in equivalent positions both spatially and in the sequences of these proteins (Figure 7). The homology between these two superfamilies has been previously reported [36].

The C-terminal domain of RNA polymerase alpha subunit and the DNA repair protein Rad51, N-terminal domain superfamilies are another pair of homologous superfamilies identified by SCOPmap. The domains in these two superfamilies have a 5-helix bundle structure (SAM domain-like fold), with one classic and one pseudo HhH motif as noted in SCOP. Members of both superfamilies have DNA-binding functions, and the observed or predicted DNA-binding surfaces are similar between the two superfamilies (Figure 7d,e). The closest representatives from each of these two superfamilies share ~32% sequence identity with each other. When the C-terminal domain of RNA polymerase alpha subunit superfamily is used as the query, all three members of this superfamily find hits to the single member of the DNA repair protein Rad51, N-terminal domain superfamily. RPS-BLAST (E-value 0.002), COMPASS (E-values ~10^-16^), and MAMMOTH (Z-scores ~9) identify these hits. The detection of both confident sequence and structure comparison hits further supports the link between these two superfamilies.

The examples discussed here are two cases among many. The examination of the complete list of potential homologs from different SCOP superfamilies is in progress.

## Conclusions

We have developed an algorithm for mapping domains within protein structures to an existing classification scheme. When applied to the SCOP database, this algorithm performs with ~95% accuracy (i.e. the correct superfamily assignment is made or no superfamily level assignment is made, as appropriate). SCOPmap produces better results than SUPERFAMILY, both in terms of overall correct assignments and in the definition of the domain boundaries of those assignments. Examination of difficult cases has demonstrated the ability of SCOPmap to make non-trivial assignments, including some domains that represent common problems associated with automatic comparison tools. SCOPmap is also capable of identifying potential evolutionary links between proteins from different SCOP superfamilies. SCOPmap should be useful to researchers interested in determining the SCOP classification of domains within newly solved protein structures. Furthermore, SCOPmap can be modified to perform similar mapping tasks within other protein classification databases. An additional potential use of the algorithm would be as an internal check in the preparation of new classifications or the maintenance and updating of existing classifications. Reliable methods for automatic updates to existing classification schemes become increasingly important with the rapid growth in sequence and structure database size.

## Methods

### Mapping strategy of the SCOPmap algorithm

#### General strategy

The purpose of SCOPmap is to assign domains within protein structures to the SCOP classification at the broadest level of homology, i.e. the SCOP superfamily level. The general strategy is to combine the results of several existing sequence and structure comparison tools to determine superfamily assignments as well as domain boundaries. Because the basis for identifying relationships between proteins varies between the different comparison tools, this combinatorial approach is expected to perform better than a single comparison tool alone. Furthermore, an approach utilizing multiple comparison tools is consistent with the conclusions reached by Novotny *et al*. from an analysis of several fold comparison servers [37].

There are three main steps in this mapping strategy. First, hits are identified between the query protein and proteins with known SCOP assignments using several existing comparison tools. Next, the results of those comparison tools are used to determine the appropriate SCOP superfamily level assignment for domains within the query. Assignments are made by a consensus-like method in which more reliable comparison tools are given preference. Finally, the algorithm uses the results of the comparison tools to define the boundaries of the domain assignments by identifying the longest non-overlapping segments.

#### Library of representative SCOP domains

A subset of SCOP domains with less than 40% identity to each other was downloaded from the ASTRAL [38,39] database. This set contains domains from the all alpha proteins, all beta proteins, alpha and beta proteins (a+b and a/b), multi-domain proteins, membrane and cell surface proteins and peptides, and small proteins classes of SCOP. Domains from the coiled coil proteins class were manually added to the library. In this paper, results using two different SCOP libraries are discussed. The library based on SCOP v1.61 contains 4813 domains from 1110 SCOP superfamilies, while the library based on SCOP v1.63 contains 5265 domains from 1232 superfamilies. Each library includes at least one representative of each SCOP superfamily.

#### Set of representative query chains

Input for SCOPmap is a list of PDB [40] identifiers. Each chain in these structures is considered as a separate query. The BLASTCLUST program (I. Dondoshansky and Y. Wolf, unpublished; ) is used for preliminary clustering of all chains at 95% sequence identity and 95% length coverage. A representative set of query chains is constructed from the first member of each BLASTCLUST cluster, excluding chains fewer than 20 residues in length. Chains less than 20 residues in length are designated as fragments and are ignored by SCOPmap.

### Mapping step 1: identifying hits between query and library domains using existing comparison methods

The gapped BLAST [41], RPS-BLAST[42], PSI-BLAST [41], COMPASS [43], MAMMOTH [19], and DaliLite [44] tools are used in SCOPmap. The first four of these are sequence comparison tools and are listed in order of increasing sensitivity to remote homologs: a query sequence against a database of sequences (gapped BLAST), a query sequence against a database of profiles (RPS-BLAST), a query profile against a database of sequences (PSI-BLAST), and a query profile against a database of profiles (COMPASS). The two structure comparison tools used are the MAMMOTH and DaliLite algorithms. Additionally, SCOPmap includes two tools which incorporate elements of both sequence and structure comparisons: correlation of conservation patterns and the agreement of pairwise alignments produced by structure comparison tools (DaliLite or MAMMOTH) with those produced by sequence comparison tools (gapped BLAST, RPS-BLAST, or PSI-BLAST). Thus, similarities between proteins are identified using eight different comparison methods, which are described in detail below.

#### Method 1) gapped BLAST [41]: query sequence against database of sequences

Gapped BLAST is run for each representative query sequence against sequences of all chains from PDB structures in SCOP (37,007 sequences in SCOP v1.61; 41,066 sequences in SCOP v1.63). The criteria for an accepted BLAST hit are an E-value ≤ 0.005 and coverage of all but 10 residues at each end of both the query and database sequences. Hits are also accepted if the query and library sequences are at least 80% identical and all but 10 residues at each end of the query sequence are covered by the alignment, irrespective of E-value. Because the database sequences used for gapped BLAST are complete chains, the accepted hits are then converted from library chains to library domains according to the SCOP-defined domain boundaries of those library sequences. This conversion is not necessary for accepted hits from the other seven comparison methods since the library representatives in those methods are domains rather than complete chains. For all query chains with accepted BLAST hits, superfamily assignment is based solely on the BLAST results and no other comparison tools are used. All query chains with no BLAST hits passing the described criteria are submitted to each of the remaining methods.

#### Method 2) RPS-BLAST [42]: query sequence against database of profiles

RPS-BLAST is run for the query sequence against a database of profiles for the library of representative SCOP domains. Profiles were constructed for each library domain by running PSI-BLAST against the non-redundant database for 5 iterations or until convergence with an E-value cutoff of 0.005. The criteria for an accepted RPS-BLAST hit are an E-value ≤ 0.005 and coverage of all but 10 residues at each end of the library domain.

#### Method 3) PSI-BLAST [41]: query profile against database of sequences

A profile for the query sequence is constructed by running PSI-BLAST against the non-redundant protein database for 5 iterations or until convergence with an E-value cutoff of 0.001. This profile is subsequently used as an input for a PSI-BLAST search against a database of all SCOP domain sequences (42465 domain sequences in SCOP v1.61; 47013 domain sequences in SCOP v1.63). The criteria for an accepted PSI-BLAST hit are an E-value ≤ 10^-4 ^and coverage of all but 10 residues at each end of the SCOP domain database sequence.

#### Method 4) COMPASS [43]: query profile against database of profiles

The profiles for the query (constructed in the PSI-BLAST step) and the SCOP library domains (constructed in the RPS-BLAST step) are prepared for COMPASS by: 1) deleting all columns with gaps in the query sequence, 2) removing all sequences identical to the query, and 3) retaining only 1 copy of any sequences in the profile that have greater than 97% identity. COMPASS is then run for the query profile against each of the SCOP library domain profiles. Accepted COMPASS hits have an E-value ≤ 10^-10 ^and coverage of all but 10 residues at each end of the library domain.

#### Method 5) MAMMOTH [19]: query structure against database of structures

The query structure is compared to each library domain structure via MAMMOTH. For each query-library domain pair, the MAMMOTH Z-score (Z_M_) and the normalized BLOSUM [45] score for the pairwise alignment made by MAMMOTH (BS_M_) are calculated. MAMMOTH hits are accepted if they meet all of the following criteria:

1) Z_M _≥ 4.0;

2) coverage of ≥50% of the library domain;

3) (BS_M _≥ 0.3) or (BS_M _≥ Z_M_^-1/2 ^- 0.24) or (Z_M _≥ 22.0).

For hits meeting only the first two criteria, the COMPASS E-value (CE_M_) is calculated for the two domains, with the alignment of the two profiles guided by the pairwise alignment made by MAMMOTH. Thus, additional accepted hits are identified that pass the following criteria: Z_M _≥ 4.0, coverage of ≥ 50% of the library domain, and CE_M _≤ 1.0. The cutoffs for accepted hits were determined based on the MAMMOTH Z-score (Z_M_), BLOSUM score (BS_M_), and COMPASS E-value (CE_M_) of 106,310 randomly chosen pairs of SCOP domains from SCOP v1.61. Approximately 1/3 of these pairs of domains belong to the same SCOP superfamily while the remaining 2/3 of the pairs belong to different SCOP superfamilies.

#### Method 6) DaliLite [44]: query structure against library structure comparisons

Additional structure comparisons are performed for queries with a segment of 20 residues or longer that did not correspond to an accepted MAMMOTH hit. Query-library domain pairs for which BS_M _≥ -0.01*Z_M _- 0.03, Z_M _> 0, and the pairwise alignment made by MAMMOTH covered at least 40% of the library domain are identified. If more than 200 query-library domain pairs met these criteria, only the 200 query-library domain pairs with the highest Z_M _scores are selected. If no pairs meet these criteria, the 50 query-library domain pairs with the highest Z_M _scores are identified. The score cutoffs for selecting pairs for comparison via DaliLite were determined by evaluating the MAMMOTH Z-scores (Z_M_) and BLOSUM scores (BS_M_) for randomly chosen pairs of SCOP domains that pass the DaliLite score cutoffs (see below) but fail the MAMMOTH score cutoffs (see above). The threshold was chosen by determining the score cutoffs that would identify the most number of pairs passing the DaliLite cutoffs and the fewest pairs failing the DaliLite cutoffs, thereby maximizing the number of potential accepted hits while minimizing the overall computation time required. DaliLite structure comparison is performed for each of the selected query-library domain pairs, and the DaliLite Z-score (Z_D_) and the normalized BLOSUM score for the pairwise alignment made by DaliLite (BS_D_) are calculated. Hits are accepted if they meet one of the following sets of criteria:

1) Z_D _≥ 4.0, BS_D _≥ -0.01*Z_D _+ 0.15, and coverage of ≥50% of the library domain;

2) BS_D _≥ 0.3 and coverage of ≥50% of the library domain;

3) Z_D _≥ 14.0 and coverage of ≥50% of the library domain.

The cutoffs for accepted hits were determined based on the DaliLite Z-score (Z_D_) and BLOSUM score (BS_D_) of 4000 randomly chosen pairs of SCOP domains from SCOP v1.61, where half of these pairs belong to the same superfamily and half of the pairs belong to different superfamilies.

#### Method 7) CSV: correlation of conservation patterns

Because homologous domains often have similar conservation patterns, the degree of correlation between the conservation patterns of two domains can be used for remote homolog detection. Distant homologs typically display drastically diminished overall sequence similarity. Thus, such cases of remote homology are more likely to be identified by conservation pattern analysis, which considers only the most conserved residues, rather than by typical sequence comparison methods, which are highly dependent on overall sequence similarity. Conservation scores for query-library domain pairs are calculated by two methods: using a conservation substitution matrix and using the COMPASS algorithm.

The query-library domain pairs selected for conservation pattern comparison are determined based on the results of the DaliLite pairwise comparisons in the previous method. The correlation of conservation patterns are calculated for all query-library domain pairs with Z_D _≥ 4.0, or for the 20 pairs with highest DaliLite Z-score (Z_D _≥ 2.0 required) if no pairs have DaliLite Z-score ≥ 4. Only pairs for which the library domain profile (constructed for the RPS-BLAST step and modified for the COMPASS step) contains 5 or more sequences are considered. The AL2CO algorithm [46] (window size 3) is used to calculate the entropy-based conservation index for each position in the query profile and in the library domain profile. DaliLite-aligned positions scoring in the top 25% of either profile are selected, henceforth referred to as the chosen positions.

Any two given positions from the profiles of the query and library domains can be compared to determine their similarity in terms of conservation patterns. The degree of correlation between those conservation patterns is referred to as the position-pair conservation score. For example, if both positions are highly conserved, the position-pair conservation score for that specific pair will be high. Conversely, if one position is highly conserved while the amino acid distribution in the other position is random, the position-pair conservation score will be low. In the first scoring system, position-pair conservation scores are determined based on the entropy-based conservation indices for the chosen positions with a conservation substitution matrix used as a scoring matrix. Then, the scoring matrix-based conservation score is calculated for the query-library domain pair by:

CSV_cons,D _= [S_n _- S_rand_]/ [(S_1_+S_2_)/2 - S_rand_],

where S_n _is the sum of position-pair conservation scores of the aligned query positions vs. library domain positions ("chosen positions" only, see above), S_1 _is the sum of position-pair conservation scores of the chosen query positions against themselves (query positions vs. query positions), S_2 _is the sum of position-pair conservation scores of the chosen library domain positions against themselves (library domain positions vs. library domain positions), and S_rand _is the sum of position-pair conservation scores of the chosen positions for all-against-all query positions vs. library domain positions normalized over length.

A COMPASS-based conservation score is also calculated for each query-library domain pair. In this scoring system, a COMPASS-based position-pair score, which describes the similarity between any two given positions, is determined based on the methodology introduced in the COMPASS method [43]. Then, the COMPASS-based conservation score for the query-library domain pair is calculated by:

CSV_compass,D _= [CS_n _- CS_rand_]/ [(CS_1_+CS_2_)/2 - CS_rand_],

where CS_n _is the sum of COMPASS-based position-pair scores of the aligned query positions vs. library domain positions ("chosen positions" only, see above), CS_1 _is the sum of COMPASS-based position-pair scores of the chosen query positions against themselves (query positions vs. query positions), CS_2 _is the sum of COMPASS-based position-pair scores of the chosen library domain positions against themselves (library domain positions vs. library domain positions), and CS_rand _is the sum of COMPASS-based position-pair scores of the chosen positions for all-against-all query positions vs. library domain positions normalized over length.

Conservation score hits are accepted if they meet one of the following sets of criteria:

1) CSV_cons,D _≥ 0.1 and Z_D _≥ 5;

2) CSV_cons,D _≥ 0.25 and Z_D _≥ 2;

3) CSV_compass,D _≥ 0.4 and Z_D _≥ 5;

4) CSV_compass,D _≥ 0.5 and Z_D _≥ 2.

These cutoffs for accepting hits were determined based on the CSV_cons,D _scores, CSV_compass,D _scores, and DaliLite Z-scores of 4000 randomly chosen pairs of SCOP domains from SCOP v1.61.

In cases for which the DaliLite program produces no output, conservation pattern analysis is performed using pairwise alignment produced by MAMMOTH instead of FSSP alignments. The conservation analysis is done for the query-library domain pairs that would have otherwise been submitted to the DaliLite algorithm for structural comparison (see above). Only those residue pairs in which the Cα atoms are located within 4Å, which are indicated by an asterisk (*) by the MAMMOTH algorithm, are considered. Again, a window size of 3 is used in the AL2CO program and only the top scoring 25% of positions are used for calculating the conservation scores. Matrix-based and COMPASS-based conservation scores are calculated as described above. Conservation score hits based on MAMMOTH alignments are accepted if they meet one of the following sets of criteria:

1) CSV_cons,M _≥ 0.3 and Z_M _≥ 4;

2) CSV_compass,M _≥ 0.4 and Z_M _≥ 4

These cutoffs for accepting hits were determined based on the CSV_cons,M _scores, CSV_compass,M _scores, and MAMMOTH Z-scores of 2000 randomly chosen pairs of SCOP domains from SCOP v1.61.

#### Method 8) agreement of DaliLite or MAMMOTH alignments with gapped BLAST, RPS-BLAST, or PSI-BLAST alignments

Remote evolutionary links between protein domains can be gleaned using a combination of sequence and structural information, even when neither of these methods alone is capable of providing convincing evidence for common descent. In this method, the degree of correlation between a pairwise alignment made by DaliLite and alignments made by the sequence comparison methods is determined so that DaliLite can be used to evaluate potential hits from BLAST, RPS-BLAST, or PSI-BLAST. For any query-library domain pair with Z_D _> 0 and BLAST, PSI-BLAST, or RPS-BLAST E-value ≤ 100, the number of correctly aligned residues (N_ali_) in the sequence alignment is calculated using the DaliLite alignment as a reference. Hits are accepted for which Z_D _> 0, E-value ≤ 100, and N_ali _≥ 15. These cutoffs were determined based on the DaliLite Z-scores, E-values, and number of equivalently aligned residues from 1000 randomly chosen pairs of SCOP domains from SCOP v1.61. If an error occurs while running DaliLite for the query domain, agreement of the MAMMOTH alignment and BLAST, RPS-BLAST, or PSI-BLAST alignments is instead calculated for the same potential hits. In these cases, hits are accepted for which Z_M _> 2.0, E-value ≤ 100, and N_ali _≥ 15. These cutoffs were determined based on the MAMMOTH Z-scores, E-values, and number of equivalently aligned residues from 1000 randomly chosen pairs of SCOP domains from SCOP v1.61.

### Mapping step 2: assigning domains from query chains to SCOP superfamilies

Accepted hits from the sequence and structure comparison methods are mapped onto the query chain and domains within the chain are then assigned to SCOP superfamilies. In cases where accepted hits from multiple SCOP superfamilies mapped to the same region of the query chain, SCOPmap attempts to choose only one correct SCOP superfamily assignment. If the overlap between two different SCOP superfamily representatives covers <50% of both domains, the conflict is resolved by the domain boundary definition (see "Mapping Step 3" below). Otherwise, SCOPmap attempts to determine which SCOP superfamily among the accepted hits is most likely to be the correct assignment.

First, for each of two conflicting assignments, all accepted hits that overlap by at least 75% and are from the same SCOP superfamily are identified. For each set of accepted hits (one set corresponding to each of the conflicting SCOP superfamilies), the number of methods that identified accepted hits to that SCOP superfamily is determined. If one SCOP superfamily is found by more methods than the other SCOP superfamily, the assignment with hits from the greater number of methods is accepted as correct. If both SCOP superfamilies are identified by an equal number of methods, the priority of those methods is used to choose the correct SCOP superfamily. The methods are ranked by reliability, which was subjectively determined based primarily on the observed number of false positives accepted by a given method during SCOPmap development. Priority rankings are as follows: BLAST > RPS-BLAST or PSI-BLAST > MAMMOTH or DaliLite > COMPASS > conservation pattern correlation or agreement of DaliLite and sequence method alignments. If both SCOP superfamilies are found by methods with equivalent priorities, the Z-scores and E-values of the hits are evaluated. If only one of the two conflicting SCOP superfamilies has E-values from any sequence comparison method below 10^-10 ^or Z-scores (Z_M _or Z_D_) above 14.0, that SCOP superfamily assignment is accepted as correct. If a SCOP superfamily assignment has still not been made, the domain assignments to that query chain are flagged as unresolved. Of the 4580 tweaking set domains (see Results), only 25 domains (0.5%) were unassigned due to unresolved choice between conflicting SCOP superfamilies. The results obtained by inverting the order of these two steps (e.g. first comparing E-values and Z-scores, and then considering priority rankings of the eight methods) were also evaluated. There were no cases where the inverted order gave additional correct assignments, and there was a small number of cases that could be resolved by the original strategy but not by the inverted strategy. Thus, the methodology described above is used for choosing between conflicting superfamily assignments.

### Mapping step 3: defining boundaries of domain assignments

Domain boundary definitions are assigned by identifying the longest non-overlapping domain assignments, with priority given to assignments made by structure comparison methods. First, DaliLite is run for all query-library domain pairs found by MAMMOTH, and the DaliLite range is used in place of the MAMMOTH range unless there is an error in the DaliLite output. Then, ranges of accepted hits are given priority rankings based on which method determined the range of that hit. DaliLite ranges have highest priority, followed by MAMMOTH ranges, and then all sequence comparison method ranges. The longest non-overlapping segments with the highest priority rankings are then identified. A 3-residue cushion for overlap is allowed. Overlapping domains for which boundaries cannot be reconciled within 3 residues are flagged as unresolved. Of 4580 tweaking set domains, only 3 domains (0.1%) were unassigned due to unresolved domain boundary definition.

### Assignments at the SCOP fold level

For query chains with a segment at least 20 residues in length which is not assigned to a SCOP superfamily, mapping at the SCOP fold level is attempted. In the SCOPmap algorithm, MAMMOTH is run comprehensively against the library of representative structures. Therefore, no additional comparisons must be made in order for fold level assignments to be determined. For this reason, MAMMOTH is used for fold level assignments rather than DaliLite, which is typically run against less than 5% of the library domains. The single criterion for potential SCOP fold assignment is a MAMMOTH Z-score > 10. Fold level assignments are made by selecting the hit to an unmapped region with the highest MAMMOTH Z-score (>10) that also covers at least 50% of the library domain. The fold level Z-score cutoff was determined based on the MAMMOTH Z-scores of 106,310 randomly chosen pairs of SCOP domains from SCOP v1.61. These same pairs of domains were used for determining the superfamily assignment cutoffs (see above). Approximately 2/3 of these pairs of domains belong to the same SCOP fold while the remaining 1/3 of the pairs belong to different SCOP folds.

### Description of test sets

SCOPmap performance was evaluated on two separate test sets. The first set is comprised of the proteins that are included in SCOP v1.63 but not in SCOP v1.61. SCOPmap was run using a library based on the previous SCOP release (v1.61), and the SCOPmap domain assignments were compared to the SCOP-defined classification in subsequent SCOP release (v1.63). This set contains 5133 SCOP-defined protein domains, but analysis of SCOPmap performance is based only on the 4580 SCOP-defined domains with evolutionary relevance: 464 low resolution structure domains, 63 peptides, 21 designed proteins, and 5 domains that were later removed from the database are intentionally excluded. The first test set was used to establish whether the score cutoffs for the individual comparison tools used by SCOPmap were strict enough to avoid false positive assignments. After first running SCOPmap for this set of domains, a false positive rate of ~1.5% was observed. The score thresholds for some of the individual comparison tools were subsequently made more strict in order to avoid all false positive assignments in this set. For example, the E-value cutoff for PSI-BLAST was changed from 5 × 10^-3 ^to 1 × 10^-4^, and the E-value cutoff for COMPASS was adjusted from 1 × 10^-4 ^to 1 × 10^-10^. Because some of the domains in this set were considered while establishing the score thresholds, the first test set is more correctly described as a "tweaking" set rather than a testing set. This set was also used for comparison to SUPERFAMILY, for which the score threshold was also chosen specifically for the purpose of precluding false positive assignments. The recommended 0.02 E-value cutoff for SUPERFAMILY, which would allow for the correct assignment of only an additional ~1% of the tweaking set domains, was not chosen due to the 4.3% false positive rate it incurs. Instead, the E-value cutoff was set at 1 × 10^-5^, the maximum value for which no false positive assignments were observed. For this comparison, the SUPERFAMILY algorithm was used with the library of SAM [47] hidden Markov models based on SCOP v1.61.

The second set of domains used to evaluate SCOPmap performance contains proteins included in SCOP v1.65 but not in SCOP v1.63. The second test set can be considered a true testing set. The testing set contains 5335 SCOP-defined protein domains, but only the 4941 SCOP-defined domains with evolutionary relevance were used for analysis of SCOPmap performance. Low resolution structures, peptides, and designed proteins were ignored. The library of SCOP representative domains used for mapping the queries in this set is based on SCOP v1.63.

### Using SCOPmap to identify homologs between SCOP superfamilies

SCOPmap can also be used to identify potentially homologous proteins that belong to different SCOP superfamilies. Detection of such homologs is accomplished with a slightly altered strategy from the mapping algorithm described above. The modified algorithm evaluates one SCOP superfamily at a time by attempting to detect potential hits to SCOP domains belonging to other superfamilies via the comparison methods described above. A set of query domains is constructed from the domains that are currently included in that SCOP superfamily (based on SCOP v1.63). As in the original mapping algorithm, the query sequences are first clustered at high sequence identity to reduce the computational time. Next, each of the 8 comparison methods described above is employed for each representative query. In the original mapping strategy, queries for which accepted hits are detected via gapped BLAST are not submitted to any of the other comparison methods. However, in this modified strategy, all comparison tools are run for all representative queries, regardless of the results of the gapped BLAST step. The output is a list of all accepted hits from each of the comparison methods to SCOP domains that do not belong to the query superfamily. All hits to SCOP domains within the query superfamily are simply ignored and excluded from the output. Finally, manual analysis of potential hits was performed for selected examples in order to evaluate the significance of those hits and to determine whether an evolutionary link is likely to exist between the two SCOP superfamilies in question.

### Program availability

The SCOPmap script and instructions for library construction are available for download at . SCOPmap results for representative PDB structures that are not included in the SCOP database are available here as well.

## Authors' contributions

SC developed the code, tested program performance, analyzed the results, and drafted the manuscript. YQ contributed to code development. SSK determined score thresholds for the individual comparison tools used. LNK proposed many additional suggestions for improving algorithm performance. NVG conceived of the study and participated in its design and coordination. All authors read and approved the final manuscript.

## Figures and Tables

**Table 1:** Results of the automatic mapping of PDB structures to SCOP superfamilies.

	*v1.61-v1.63 test set*						*v1.63-v1.65 test set*	
	
*Result*	*SCOPmap*		*SCOPmap, sequence comparison tools only*		*SUPERFAMILY*		*SCOPmap*	
	
	*# of domains*	*% of test set (bold: correct assignments)*	*# of domains*	*% of test set (bold: correct assignments)*	*# of domains*	*% of test set (bold: correct assignments)*	*# of domains*	*% of test set (bold: correct assignments)*
Assignment to correct SCOP superfamily, boundaries accurate within 10 residues	3730	**81.4%**	3507	**76.6%**	3211	**70.1%**	4136	**83.7%**

Assignment to correct SCOP superfamily, boundaries *not *accurate within 10 residues	292	**6.4%**	211	**4.6%**	662	**14.4%**	372	**7.5%**

Domain belongs to a *new *SCOP superfamily, no assignment made	284	**6.2%**	289	**6.3%**	241	**5.3%**	154	**3.1%**

Acceptable assignment, but not the same assignment as given in SCOP	13	**0.3%**	0	**0%**	71	**1.5%**	12	**0.2%**

Incorrect assignment	0	0%	0	0%	0	0%	7	0.2%

Domain belongs to an *existing *SCOP superfamily, no assignment made	261	5.7%	573	12.5%	395	8.6%	260	5.3%

**Table 2:** Results for the 1417 non-trivial assignments.

	*SCOPmap*		*SUPERFAMILY*	
	
*Result*	*# of domains*	*% of test set (bold: correct assignments)*	*# of domains*	*% of test set (bold: correct assignments)*
Assignment to correct SCOP superfamily, boundaries within 10 residues	607	**42.8%**	425	**30.0%**

Assignment to correct SCOP superfamily, boundaries not within 10 residues	252	**17.8%**	379	**26.7%**

Domain belongs to a new SCOP superfamily, no assignment made	284	**20.0%**	241	**17.0%**

Acceptable assignment, but not the same assignment as given in SCOP	13	**0.9%**	48	**3.4%**

Domain belongs to an existing SCOP superfamily, no assignment made	261	18.4%	324	22.9%

**Table 3:** Tweaking set domain assignments by increasingly sensitive comparison tools.

*Comparison Method*	*Number of Domains First Identified By This Method *[4035 mapped domains plus 50 domains that are identified but not assigned (see Table 4)]	*Average Sequence Identity Between Query and Closest Superfamily Representative*	*% of Domains Unmapped by Less Sensitive Methods that are Identified by This Method*
BLAST	3163	80.1%	69.1%
RPS-BLAST	514	41.1%	36.3%
PSI-BLAST	104	26.1%	11.5%
COMPASS	26	27.2%	3.3%
MAMMOTH	100	29.7%	12.9%
DaliLite	124	17.4%	18.4%
correlation of conservation patterns	23	11.1%	4.2%
agreement of alignments produced by DaliLite and by gapped BLAST, RPS-BLAST, or PSI-BLAST	31	12.1%	5.9%

**Table 4:** SCOPmap: Automated assignment of protein structures to evolutionary superfamlies.

*Whether Domain is Identified by at Least One Comparison Method*	*Reason Domain is Unmapped*	*Number of Domains*	*% of Unassigned Domains*	
The domain is identified by one or more methods, but is not assigned.	The boundary assigned to one domain in the query chain is extended too far and, as a result, a second domain assignment is missed.	22	8.5%	
				19.2%
	Unresolved choice between conflicting superfamilies.	28	10.7%	

Domain is not identified by any comparison tool used in SCOPmap.	DaliLite hits to superfamily representatives fail "accepted hit" cutoffs.	108	41.4%	
				80.8%
	At least one superfamily representative identified as potential hit via MAMMOTH, but DaliLite produces no output for the comparison.	16	6.1%	
		
	No superfamily representatives have MAMMOTH scores high enough to be identified as potential hits via DaliLite.	87	33.3%	

## References

[B1] Murzin AG, Brenner SE, Hubbard T, Chothia C (1995). SCOP: a structural classification of proteins database for the investigation of sequences and structures. J Mol Biol.

[B2] Orengo CA, Michie AD, Jones S, Jones DT, Swindells MB, Thornton JM (1997). CATH--a hierarchic classification of protein domain structures. Structure.

[B3] Dietmann S, Holm L (2001). Identification of homology in protein structure classification. Nat Struct Biol.

[B4] Russell RB, Barton GJ (1994). Structural features can be unconserved in proteins with similar folds. An analysis of side-chain to side-chain contacts secondary structure and accessibility. J Mol Biol.

[B5] Russell RB, Saqi MA, Sayle RA, Bates PA, Sternberg MJ (1997). Recognition of analogous and homologous protein folds: analysis of sequence and structure conservation. J Mol Biol.

[B6] Holm L, Sander C (1997). Decision support system for the evolutionary classification of protein structures. Proc Int Conf Intell Syst Mol Biol.

[B7] Matsuo Y, Bryant SH (1999). Identification of homologous core structures. Proteins.

[B8] Krissinel E, Henrick K, Kungl AJ and Kungl PJ (2003). Protein structure comparison in 3D based on secondary structure matching (SSM) followed by Ca alignment, scored by a new structural similarity function: September 3-7 2003; Vienna..

[B9] Harrison A, Pearl F, Sillitoe I, Slidel T, Mott R, Thornton J, Orengo C (2003). Recognizing the fold of a protein structure. Bioinformatics.

[B10] Getz G, Vendruscolo M, Sachs D, Domany E (2002). Automated assignment of SCOP and CATH protein structure classifications from FSSP scores. Proteins.

[B11] Gough J, Karplus K, Hughey R, Chothia C (2001). Assignment of homology to genome sequences using a library of hidden Markov models that represent all proteins of known structure. J Mol Biol.

[B12] Jing H, Takagi J, Liu JH, Lindgren S, Zhang RG, Joachimiak A, Wang JH, Springer TA (2002). Archaeal surface layer proteins contain b propeller, PKD, and b helix domains and are related to metazoan cell surface proteins. Structure (Camb).

[B13] Loris R, Marianovsky I, Lah J, Laeremans T, Engelberg-Kulka H, Glaser G, Muyldermans S, Wyns L (2003). Crystal structure of the intrinsically flexible addiction antidote MazE. J Biol Chem.

[B14] Vaughn JL, Feher V, Naylor S, Strauch MA, Cavanagh J (2000). Novel DNA binding domain and genetic regulation model of Bacillus subtilis transition state regulator abrB. Nat Struct Biol.

[B15] Nishino T, Komori K, Ishino Y, Morikawa K (2003). X-ray and biochemical anatomy of an archaeal XPF/Rad1/Mus81 family nuclease: similarity between its endonuclease domain and restriction enzymes. Structure (Camb).

[B16] Senda T, Yamada T, Sakurai N, Kubota M, Nishizaki T, Masai E, Fukuda M, Mitsuidagger Y (2000). Crystal structure of NADH-dependent ferredoxin reductase component in biphenyl dioxygenase. J Mol Biol.

[B17] Heikinheimo P, Helland R, Leiros HK, Leiros I, Karlsen S, Evjen G, Ravelli R, Schoehn G, Ruigrok R, Tollersrud OK, McSweeney S, Hough E (2003). The structure of bovine lysosomal a-mannosidase suggests a novel mechanism for low-pH activation. J Mol Biol.

[B18] Hondoh H, Kuriki T, Matsuura Y (2003). Three-dimensional structure and substrate binding of Bacillus stearothermophilus neopullulanase. J Mol Biol.

[B19] Ortiz AR, Strauss CE, Olmea O (2002). MAMMOTH (matching molecular models obtained from theory): an automated method for model comparison. Protein Sci.

[B20] Bauer S, Kemter K, Bacher A, Huber R, Fischer M, Steinbacher S (2003). Crystal structure of Schizosaccharomyces pombe riboflavin kinase reveals a novel ATP and riboflavin-binding fold. J Mol Biol.

[B21] Meinhart A, Alonso JC, Strater N, Saenger W (2003). Crystal structure of the plasmid maintenance system e/z: functional mechanism of toxin z and inactivation by e2z2 complex formation. Proc Natl Acad Sci U S A.

[B22] Alexander JM, Nelson CA, van Berkel V, Lau EK, Studts JM, Brett TJ, Speck SH, Handel TM, Virgin HW, Fremont DH (2002). Structural basis of chemokine sequestration by a herpesvirus decoy receptor. Cell.

[B23] Yankovskaya V, Horsefield R, Tornroth S, Luna-Chavez C, Miyoshi H, Leger C, Byrne B, Cecchini G, Iwata S (2003). Architecture of succinate dehydrogenase and reactive oxygen species generation. Science.

[B24] Suresh S, Turley S, Opperdoes FR, Michels PA, Hol WG (2000). A potential target enzyme for trypanocidal drugs revealed by the crystal structure of NAD-dependent glycerol-3-phosphate dehydrogenase from Leishmania mexicana. Structure Fold Des.

[B25] Korolev SV, Dementieva IS, Christendat D, Edwards A, Joachimiak A Structural Similarities of Mth1747 Hypothetical Protein from Methanobacterium Thermoautotrophicum with 3-Hydroxyacid Dehydrogenases. In preparation.

[B26] Lee BI, Chang C, Cho SJ, Eom SH, Kim KK, Yu YG, Suh SW (2001). Crystal structure of the MJ0490 gene product of the hyperthermophilic archaebacterium Methanococcus jannaschii, a novel member of the lactate/malate family of dehydrogenases. J Mol Biol.

[B27] Sanchez JF, Hoh F, Strub MP, Aumelas A, Dumas C (2002). Structure of the cathelicidin motif of protegrin-3 precursor: structural insights into the activation mechanism of an antimicrobial protein. Structure (Camb).

[B28] Bode W, Engh R, Musil D, Thiele U, Huber R, Karshikov A, Brzin J, Kos J, Turk V (1988). The 2.0 A X-ray crystal structure of chicken egg white cystatin and its possible mode of interaction with cysteine proteinases. Embo J.

[B29] Yasutake Y, Watanabe S, Yao M, Takada Y, Fukunaga N, Tanaka I (2002). Structure of the monomeric isocitrate dehydrogenase: evidence of a protein monomerization by a domain duplication. Structure (Camb).

[B30] Wallon G, Kryger G, Lovett ST, Oshima T, Ringe D, Petsko GA (1997). Crystal structures of Escherichia coli and Salmonella typhimurium 3-isopropylmalate dehydrogenase and comparison with their thermophilic counterpart from Thermus thermophilus. J Mol Biol.

[B31] Tao Y, Farsetta DL, Nibert ML, Harrison SC (2002). RNA synthesis in a cage--structural studies of reovirus polymerase l3. Cell.

[B32] Georgiadis MM, Jessen SM, Ogata CM, Telesnitsky A, Goff SP, Hendrickson WA (1995). Mechanistic implications from the structure of a catalytic fragment of Moloney murine leukemia virus reverse transcriptase. Structure.

[B33] Lamoureux JS, Stuart D, Tsang R, Wu C, Glover JN (2002). Structure of the sporulation-specific transcription factor Ndt80 bound to DNA. Embo J.

[B34] Zeth K, Ravelli RB, Paal K, Cusack S, Bukau B, Dougan DA (2002). Structural analysis of the adaptor protein ClpS in complex with the N-terminal domain of ClpA. Nat Struct Biol.

[B35] Kohno T, Sasaki T, Kobayashi K, Fainzilber M, Sato K (2002). Three-dimensional solution structure of the sodium channel agonist/antagonist d-conotoxin TxVIA. J Biol Chem.

[B36] Nagano N, Orengo CA, Thornton JM (2002). One fold with many functions: the evolutionary relationships between TIM barrel families based on their sequences, structures and functions. J Mol Biol.

[B37] Novotny M, Madsen D, Kleywegt GJ (2004). Evaluation of protein fold comparison servers. Proteins.

[B38] Brenner SE, Koehl P, Levitt M (2000). The ASTRAL compendium for protein structure and sequence analysis. Nucleic Acids Res.

[B39] The ASTRAL Compendium for Sequence and Structure Analysis. http://astral.berkeley.edu.

[B40] Berman HM, Westbrook J, Feng Z, Gilliland G, Bhat TN, Weissig H, Shindyalov IN, Bourne PE (2000). The Protein Data Bank. Nucleic Acids Res.

[B41] Altschul SF, Madden TL, Schaffer AA, Zhang J, Zhang Z, Miller W, Lipman DJ (1997). Gapped BLAST and PSI-BLAST: a new generation of protein database search programs. Nucleic Acids Res.

[B42] Marchler-Bauer A, Anderson JB, DeWeese-Scott C, Fedorova ND, Geer LY, He S, Hurwitz DI, Jackson JD, Jacobs AR, Lanczycki CJ, Liebert CA, Liu C, Madej T, Marchler GH, Mazumder R, Nikolskaya AN, Panchenko AR, Rao BS, Shoemaker BA, Simonyan V, Song JS, Thiessen PA, Vasudevan S, Wang Y, Yamashita RA, Yin JJ, Bryant SH (2003). CDD: a curated Entrez database of conserved domain alignments. Nucleic Acids Res.

[B43] Sadreyev R, Grishin N (2003). COMPASS: a tool for comparison of multiple protein alignments with assessment of statistical significance. J Mol Biol.

[B44] Holm L, Sander C (1995). Dali: a network tool for protein structure comparison. Trends Biochem Sci.

[B45] Henikoff S, Henikoff JG (1992). Amino acid substitution matrices from protein blocks. Proc Natl Acad Sci U S A.

[B46] Pei J, Grishin NV (2001). AL2CO: calculation of positional conservation in a protein sequence alignment. Bioinformatics.

[B47] Karplus K, Barrett C, Hughey R (1998). Hidden Markov models for detecting remote protein homologies. Bioinformatics.

[B48] Kraulis PJ (1991). MOLSCRIPT: a program to produce both detailed and schematic plots of protein structures. Journal of Applied Crystallography.

[B49] Cramer P, Larson CJ, Verdine GL, Muller CW (1997). Structure of the human NF-kB p52 homodimer-DNA complex at 2.1 A resolution. Embo J.

[B50] Leijonmarck M, Liljas A (1987). Structure of the C-terminal domain of the ribosomal protein L7/L12 from Escherichia coli at 1.7 A. J Mol Biol.

[B51] Kobayashi K, Sasaki T, Sato K, Kohno T (2000). Three-dimensional solution structure of w-conotoxin TxVII, an L-type calcium channel blocker. Biochemistry.

[B52] Peapus DH, Chiu HJ, Campobasso N, Reddick JJ, Begley TP, Ealick SE (2001). Structural characterization of the enzyme-substrate, enzyme-intermediate, and enzyme-product complexes of thiamin phosphate synthase. Biochemistry.

[B53] Knochel T, Pappenberger A, Jansonius JN, Kirschner K (2002). The crystal structure of indoleglycerol-phosphate synthase from Thermotoga maritima. Kinetic stabilization by salt bridges. J Biol Chem.

[B54] Benoff B, Yang H, Lawson CL, Parkinson G, Liu J, Blatter E, Ebright YW, Berman HM, Ebright RH (2002). Structural basis of transcription activation: the CAP-a CTD-DNA complex. Science.

[B55] Aihara H, Ito Y, Kurumizaka H, Yokoyama S, Shibata T (1999). The N-terminal domain of the human Rad51 protein binds DNA: structure and a DNA binding surface as revealed by NMR. J Mol Biol.

